# pH-responsive supramolecular switch of a rationally designed dipyrroethene-based chromophore†

**DOI:** 10.1039/d4sc07016j

**Published:** 2024-12-16

**Authors:** Debasish Mandal, Abani Sarkar, Kanhu Charan Behera, Mangalampalli Ravikanth

**Affiliations:** a Department of Chemistry, Indian Institute of Technology Bombay Powai Mumbai-400076 India ravikanth@iitb.ac.in

## Abstract

Herein, we present a strategy to access a novel class of pH-responsive, dual-state emissive (DSE), highly fluorescent pyrrole-based chromophores *via* diformylation of dipyrroethenes (DPE) followed by condensation with various aniline derivatives. The DPE-based chromophores exhibit a large Stokes shift and maintain good fluorescence quantum yields. Remarkably, these chromophores demonstrate reversible colourimetric changes and a fluorometric ‘on–off–on’ switch in response to pH variations. Various spectroscopic techniques, optical microscopy, X-ray crystallography, and computational studies revealed that the synthesized molecules adopt a two-dimensional conformation due to the presence of strong π⋯π stacking and hydrogen bonding interactions, allowing them to function as flexible molecular hosts. Under acidic conditions, selective protonation of imine bonds and subsequent complexation with the counter anion enhance the host–guest interactions, resulting in a stable three-dimensional supramolecular structure. Notably, the reversibility of these molecules under basic conditions showcases the robustness and potential applications of these chromophores in various fields, ranging from the design of finely tuned pH-responsive degradable polymers to self-healing materials, as well as sensing and molecular devices.

## Abstract 2

We report the design and synthesis of a novel class of dual-state emissive pyrrole-based chromophores and their use as pH-responsive supramolecular switches.

## Graphical abstract

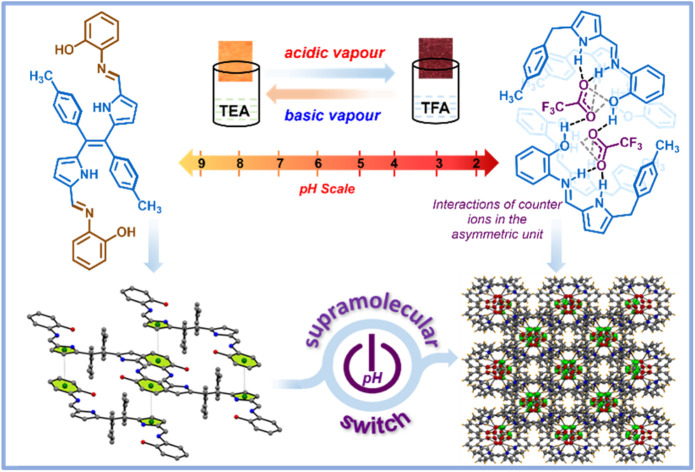

## 1. Introduction

Stimuli-responsive control over the properties of self-organized supramolecular architectures is indeed crucial towards the fabrication of smart molecular materials and devices.^1*a*,*e*^ One approach to this end is the supramolecular switch, which implies the reversible structural swapping ability of complexes from the molecular to the supramolecular level *via* non-covalent interactions like the hydrophobic effect, electrostatic interactions, hydrogen bonding, van der Waals interactions *etc.*^1^ Therefore, the supramolecular species can comprise two or more self-assembled units, whose association can be transiently controlled by various external stimuli such as light, acid, base, chemical effectors *etc.*^2–6^ In this context, supramolecular switch phenomena find myriads of currently relevant practical applications ranging from artificial molecular machines^7,8^ to supramolecular drug delivery systems^9^ and control-level catalysis.^10^ Moreover, the design and development of such a system by incorporating pH-responsive^11^ dual-state emissive small molecule-based chromophores can be a potential tool to track multiple physiological processes such as homeostasis, cell metabolism, enzyme activity, and ion transport.^12^

On the other hand, the development of small molecule-based fluorescent chromophores with a large Stokes shift is highly desirable in the realm of photochemistry and photobiology.^13^ It has a significant implication in various scientific fields such as clinical-pathological examination, fluorescence image-guided surgery, the development of fluorescent probes for sensing, materials for optoelectronics, and so on.^14–17^ Additionally, other photophysical properties of a chromophore, like the absorption–emission profile, absorption coefficient, quantum yield, chemical properties, and photochemical stability, also play a critical role. Based on these optimal properties, several types of chromophores like rhodamine, boron dipyrromethene (BODIPY), fluorescein isothiocyanate (FITC), cyanine, coumarin, and quinoline dyes have been developed.^18–22^ However, the lower emission wavelength profile (below 550 nm) and/or small Stokes shift (less than 70 nm) of most of these dyes are the major shortcomings, which lead to the need and design of fluorophores with a larger Stokes shift.^22*b*^ The fundamental advantage of large Stokes shift relays is the minimization of spectral overlap between excitation and emission wavelengths, which enhances the signal-to-noise ratio in fluorescence-based techniques, thereby facilitating more precise and sensitive detection and imaging.^23^

**Scheme 1:**
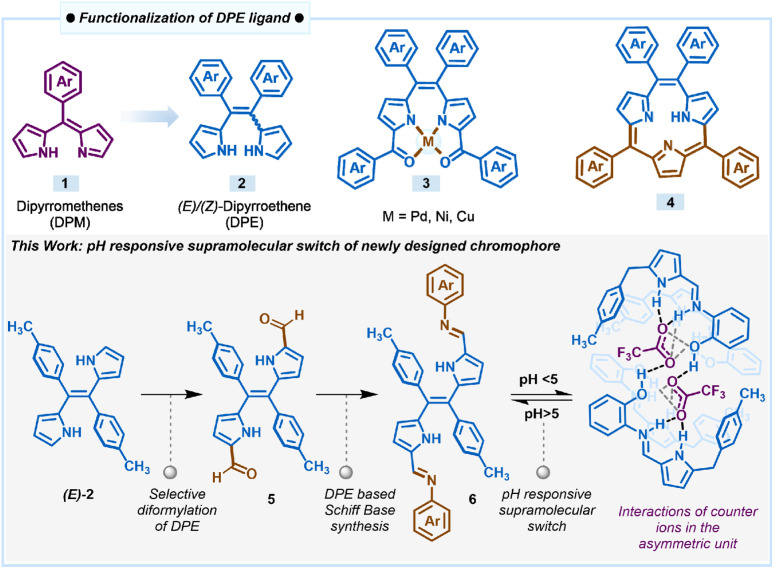
Derivatization of the DPE ligand and pH-responsive supramolecular switch of a DPE-based chromophore.

Within the context of chromophore design, functionalized pyrrole-based dyes such as BF_2_-complexes of dipyrromethenes, popularly known as BODIPYs, and their derivatives are an important class of compounds that absorb and emit in the visible-NIR region with high fluorescence quantum yields.^24,25^ This property is attributed to the extended conjugation and planarity of the dipyrromethene 1 (DPM) core containing two pyrrole rings connected by one *meso*-carbon (Scheme 1). Dipyrromethene or its saturated analogue, dipyrromethane, has been used extensively as a key precursor to prepare several types of porphyrinoids and also as the ligand for the synthesis of different types of coordination complexes.^26–29^ On the other hand, dipyrroethenes 2 (DPE), which are another class of highly stable pyrrole-based ligands that contains an additional *meso*-carbon compared to dipyrromethenes, have not been explored to the same extent as dipyrromethenes. We used dipyrroethenes or functionalized dipyrroethenes as key precursors for the synthesis of coordination complexes 3 and also for the synthesis of aromatic macrocycles such as *meso*-tetraaryl triphyrin (2.1.1)s 4 and *meso*-tetraaryl porphycenes, as well as different types of non-aromatic expanded porphyrinoids.^30–34^

Following our continuous interest in the DPE-based ligands and in the pursuit of developing pH-responsive fluorescent materials, herein, we introduced a novel class of dual-state emissive pyrrole-based chromophores 6a–6c through α,α′-diformylation of DPE 2 through the Vilsmeier–Haack reaction and condensed the diformyl DPE 5 with aniline or various *ortho*-substituted anilines (Scheme 1). The extended conjugation in the Schiff bases 6a–6c due to the introduction of the additional aryl imine bond subsequently influences the electronic nature of the molecules. We found that this modification significantly improved the optical and structural properties with a large Stokes shift (more than 120 nm) and decent fluorescence quantum yield. The X-ray structure of 6c revealed that the conjugated extended pyrrole-based system with a rigid plane provides an excellent platform for several supramolecular interactions such as intra- and inter-molecular hydrogen bonding, and π⋯π stacking, which makes these sets of ligands fluorescent even in the solid state. Interestingly, all the compounds 6a–6c showed pH-dependent rapid protonation and deprotonation at different pH, resulting in significant colourimetric and fluorometric changes attributed to the pH-responsive reversible modulation of the structural orientation. Selective protonation of the imine nitrogen of compounds when exposed to a certain acidic pH range led to the formation of a cage-like three-dimensional packing structure due to intermolecular hydrogen bonding with the corresponding counter anion. Optical microscopy images of 6c revealed notable morphological transformation in crystal shape upon exposure to TFA vapour, clearly indicating the possibility of a crystal-to-crystal transformation occurring during this phenomenon. This observation suggests that the interaction of 6c with TFA vapour induces structural rearrangement within the crystal lattice, leading to a transition between distinct crystalline phases while preserving the solid-state nature of the material. Thus, the DPE-based Schiff bases can act as a potential acidic pH sensor through the interesting pH-responsive supramolecular switch for both liquid and vapour phase acidity detection, overriding the traditional basic pH sensing ability of the many reported Schiff base ligands as described here.^34*b*^

## 2. Results and discussion

### 2.1 Synthesis and characterization of DPE-based chromophores 6a–6c

**Fig. 1:**
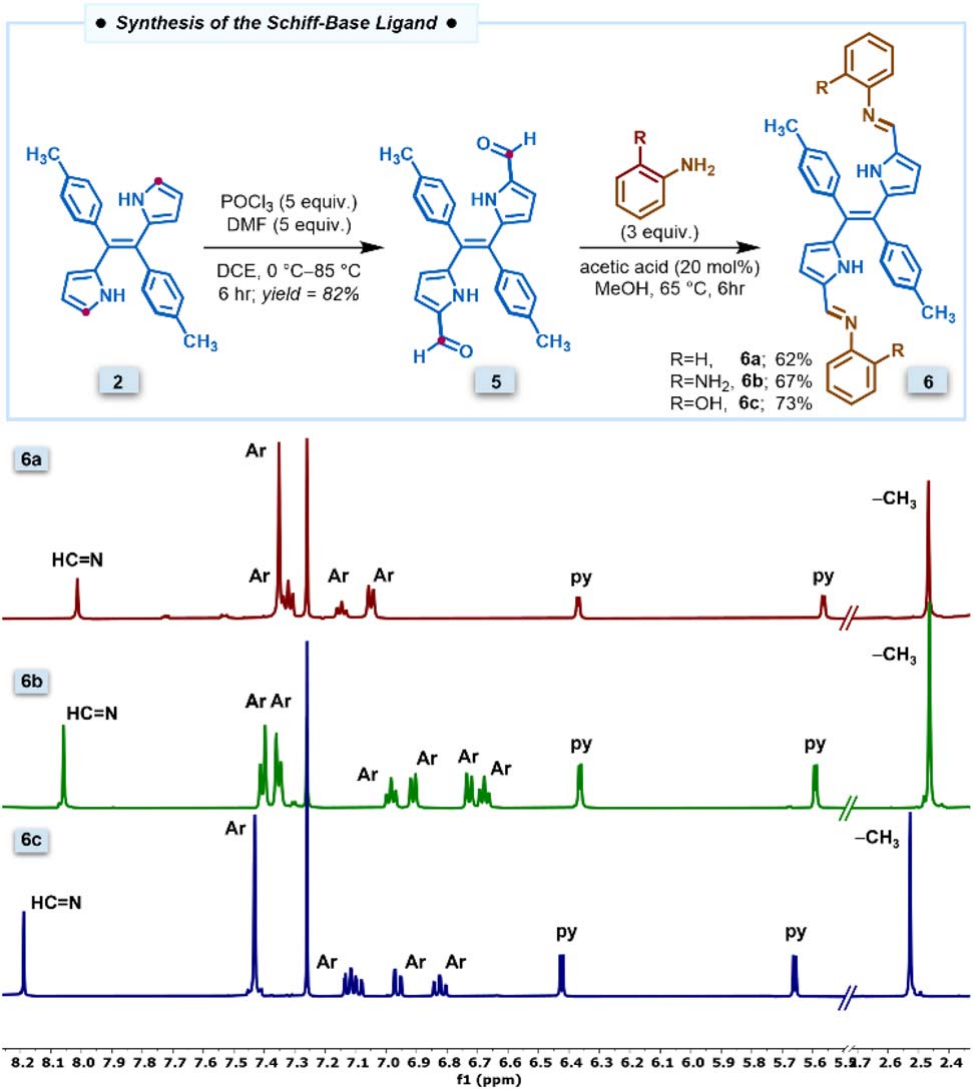
Synthesis of the Schiff base compound 6a–6c and corresponding partial ^1^H NMR spectra recorded in CDCl_3_ at room temperature.

The new class of pyrrole-based chromophores 6a–6c were prepared in two steps starting from (*E*)-dipyrroethene^35^ as shown in Fig. 1. The selective diformylation of (*E*)-dipyrroethene was carried out *via* the Vilsmeier–Haack reaction. To obtain the best yields of diformyl-DPE 5, the reactions were performed by varying different parameters such as equivalence of the POCl_3_ and the DMF, temperature, and time, and the best yields of 82% were obtained when we used five equivalents of both POCl_3_ and DMF, refluxed at 85 °C for six hours in 1,2-dichloroethane solvent (Table S1†). In the next step, the diformyl-DPE 5 was condensed with two equivalents of aniline or *ortho*-substituted anilines in the presence of a catalytic amount of glacial acetic acid in CH_3_OH for 6 h at 65 °C followed by recrystallization which afforded (*E*)-Schiff base ligands 6a–6c in 62–73% yields. The Schiff base ligands 6a–6c were characterized by HRMS, 1D & 2D NMR, ^13^C NMR, FTIR, and two complexes 6b and 6c by X-ray analysis.

The partial ^1^H NMR spectra of Schiff base ligands 6a–6c in the selected region depicted in Fig. 1 revealed that all three ligands are symmetric and exist in (*E*)-conformation. In the ^1^H NMR spectrum of 6a, the four β-protons of two pyrrole rings appeared as two sets of doublets at 5.56 ppm and 6.57 ppm; the aryl protons of the tolyl ring appeared in the region of 7.35–7.40 ppm; the –CH_3_ protons of tolyl groups appeared as a singlet at 2.46 ppm; the imine proton appeared as a singlet at 8.01 ppm whereas the aryl rings connected to imine nitrogen appeared as three sets in the region of 7.02–7.35. The compounds 6b and 6c showed similar NMR features to compound 6a. Furthermore, the resonances of compounds 6b and 6c experienced downfield shifts compared to 6a due to the involvement of the *ortho*-substituent of aryl groups in strong hydrogen bonding interactions with the imine nitrogen. Similarly, the IR stretching frequency at 1586 cm^−1^ also supports the presence of imine bonds in the IR data of 6c (Fig. S1†).

**Fig. 2:**
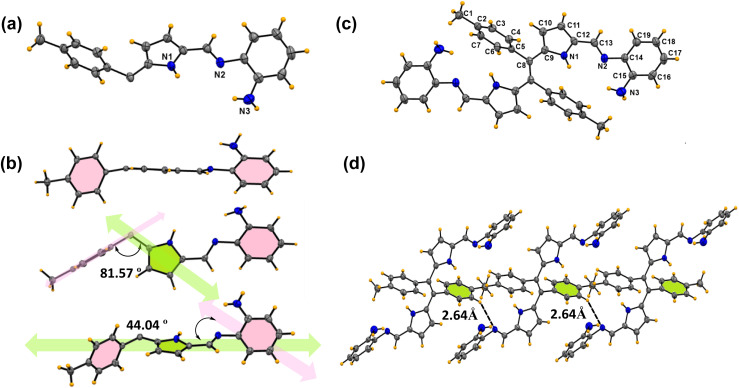
X-ray crystal structures of compound 6b. (a) Asymmetric unit (thermal ellipsoids are drawn with 50% probability). (b) Different orientations of the asymmetric unit. (c) Building block unit of compound 6b. (d) Parallel hydrogen bonding interactions between two building block units (CCDC No. of 6b2368499).

**Fig. 3:**
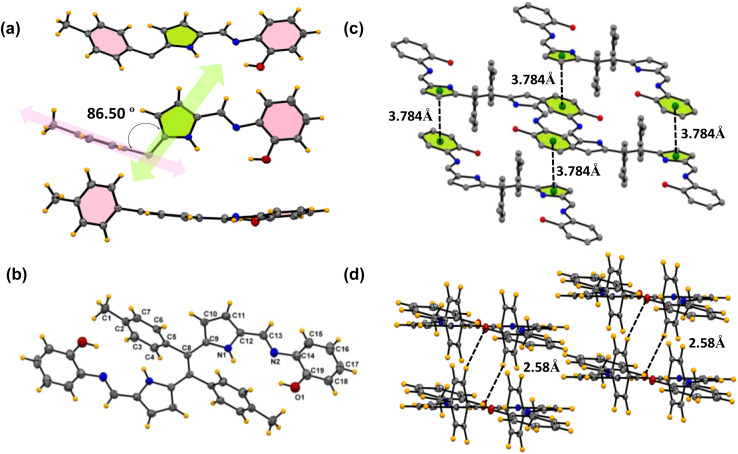
X-ray crystal structures of compound 6c. (a) Different orientations of the asymmetric unit. (b) Building block unit of compound 6c. (Thermal ellipsoids are drawn with 50% probability.) (c) The π⋯π interactions between two molecules in the unit cell. (d) Parallel intermolecular hydrogen bonding interaction between oxygen and the benzene proton (CCDC No. of 6c2345700).

Furthermore, X-ray crystallography of compounds 6b and 6c (Fig. 2 and 3) supports the formation of the desired Schiff base. X-ray analysis (Fig. 2) revealed that compound 6b was crystallized in a triclinic system with a *P*1̄ space group. The asymmetric building block unit of compound 6b is shown in Fig. 2a, and the crystal data and refinement details for compound 6b are presented in Table S2.† The asymmetric unit of compound 6b contains half of the molecule, and the molecule possesses a center of symmetry (*i*). The asymmetric unit of compound 6b consists of two aryls and one pyrrole ring connected *via* an imine bond with a bond distance of 1.281(5) Å. In particular, the tolyl ring is almost perpendicularly oriented with respect to the pyrrole ring (Fig. 2b). Interestingly, the imine bond maintains planarity with the pyrrole ring. However, both aryl rings deviate slightly from the pyrrolic ring plane. For instance, the tolyl ring maintains angles of 81.57° and 44.04° for the aniline ring. Furthermore, the crystal lattice shows strong intermolecular C⋯H–N hydrogen bond interaction with the N2 atom of the imine and H3 atom of the tolyl ring with a bond distance of 2.64(6) Å, resulting in the formation of a sheet-like structure (Fig. 2d).

Similarly, compound 6c was crystallized in a triclinic system with the *P*1̄ space group, and the structure of compound 6c is shown in Fig. 3. The crystal data and refinement details for the compounds are presented in Table S2.† The asymmetric unit and the building block unit are shown in Fig. 3a and b. The asymmetric unit consists of two aryls and one pyrrole ring, as shown in Fig. 3. Similarly to compound 6b, compound 6c also consists of one imine bond (C=N) in the asymmetric unit with a bond distance of 1.27(6) Å. In the asymmetric unit, the tolyl group and the Schiff base part are situated almost perpendicular to each other with an angle of 86.50°, but the amino phenol part lies at the same plane with respect to the imine bond and pyrrole ring, as shown in Fig. 3a. Interestingly, the unit cell of the crystal lattice shows strong intermolecular C–H⋯O hydrogen bond interaction between the O1 and H3 atoms with the bond distance of 2.58(5) Å and O1 and H4 bond distance of 2.63(6) Å (Fig. 3d). Moreover, strong intermolecular π⋯π interactions with the benzene ring (C14 C15 C16 C17 C18 C19) and pyrrole ring (C9 C10 C11 C12 N1) were observed in the system, as shown in Fig. 3c. In the packing diagram, it is found that each of the separated units is held together by intermolecular π⋯π interaction with a bond distance of 3.78 Å which allows the molecule to grow in a dimension.

**Fig. 4:**
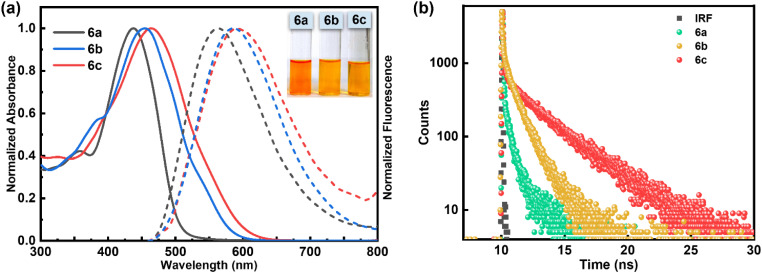
(a) Normalized absorption (solid lines) and emission spectra (dashed lines) of 6a–6c in CH_3_OH; (b) excited-state lifetime decay profiles of 6a, 6b and 6c in CH_3_OH, *λ*_Ex._ = 425 nm (for 6a), *λ*_Ex._ = 450 nm (for 6b and 6c) and *λ*_Em_ = 560 nm for 6a and *λ*_Em_ = 590 nm for 6b and 6c; concentration used: [compound] = 10 μM for absorption and 1 μM for emission.

The comparison of absorption and fluorescence spectra of Schiff base ligands 6a–6c recorded in CH_3_OH is presented in Fig. 4a, and the relevant data are tabulated in Table 1. The absorption spectra of compounds 6a–6c showed a characteristic broad absorption band in the 435–465 nm region. Upon excitation at 425–440 nm, the compounds 6a–6c exhibited an emission band in the region of 560–590 nm with a large Stokes shift (more than Δ*λ* = 120 nm; 4556–5122 cm^−1^). The observed bathochromic shift in both the absorption and emission spectra for compounds 6b and 6c compared to 6a can be attributed to the presence of an electron donating amino/hydroxyl group in the *ortho*-position of the benzene ring, which increases the electron density of the conjugated system. Interestingly, these compounds exhibit emissive properties in the solid state. The crystalline compound 6c showed a broadening in the absorption spectra, with maxima at 515 nm and a sharp emission band at 601 nm in the solid state (Fig. S2d†). Thus, Schiff base compounds 6a–6c are dual-state emissive (DSE) fluorophores with a strong emission in the solid state along with a large Stokes shift and enhanced photostability. These π-conjugated organic materials display a high degree of planarity and rigidity, which, along with possible supramolecular interactions such as π⋯π stacking and hydrogen bonding, leads to aggregation in the solid state, enabling powerful non-radiative deactivation channels.

**Table 1:** Photophysical data of compounds 6a–6c in CH_3_OH^a^

Compound	*λ*_abs_ (nm)	*λ*_em_ (nm)	Δ*ν*_st_ (cm^−1^)	Log *ε*	*Φ*	*τ*_av_ (ns)	*k*_r_ (10^9^ s^−1^)	*k*_nr_ (10^9^ s^−1^)
6a	437	563	5122	4.18	0.16	0.05	3.2	16.8
6b	455	585	4884	4.25	0.20	0.12	1.6	6.67
6c	465	590	4556	4.31	0.24	0.22	1.09	3.45

a Log *ε* (mol^−1^ dm^3^ cm^−1^) = molar extinction coefficient, *λ*_abs_ (absorption maxima), *λ*_em_ (emission maxima), Δ*ν* (Stokes shift), *Φ* (quantum yield), *τ*_av_ (average lifetime), *k*_r_ (radiative decay), and *k*_nr_ (nonradiative decay).

To measure the singlet state lifetime of the DPE-based chromophores 6a–6c, we carried out time-correlated single-photon counting measurements (TCSPC). The fluorescence decay of compounds 6a–6c was fitted to a bi-exponential function (Fig. 4b) with an average lifetime in the range of 0.05 ns to 0.22 ns (Table 1). The bi-exponential decay profile likely arises from the coexistence of multiple conformations of 6a–6c in solution, driven by dynamic C–C single bond rotations that influence their electronic environments. The fluorescence quantum yield, lifetimes, and radiative and non-radiative rate constants of all the compounds are summarized in Table 1.

### 2.2 pH-responsive properties of DPE-based chromophores 6a–6c

**Fig. 5:**
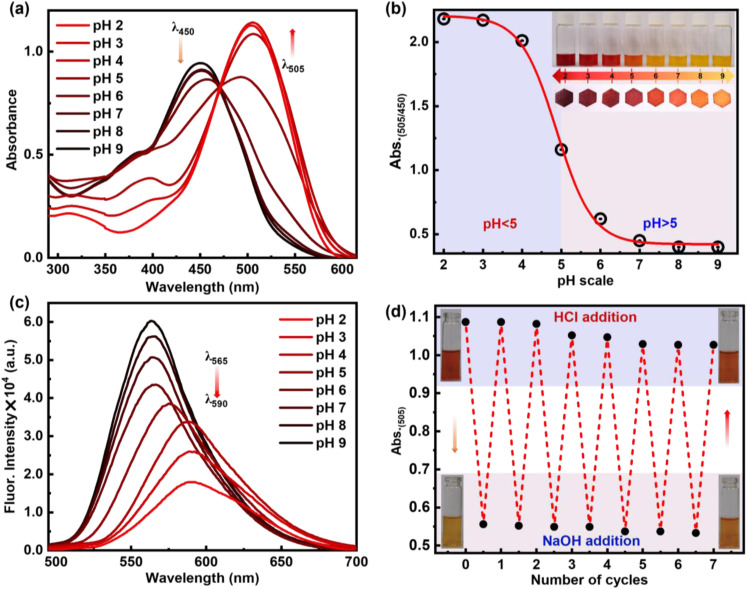
(a) Absorption spectra of 6c with respect to different pH ranging from 2–9. (b) pH titration plot of absorbance ratio (*λ*^Abs^_505_/*λ*^Abs^_450_) *vs.* pH. (c) Emission spectra of 6c at varying pH ranging from 2–9. (d) Stability, reusability and reversibility in absorbance spectral change with 6c on sequential addition of HCl and NaOH. Concentration of 6c: 10 μM for absorption and 1 μM for emission.

Schiff bases are generally regarded as highly effective colourimetric and fluorometric sensors for detecting various pH levels. This is attributed to the structure–function properties of these molecules, which enable them to respond sensitively to changes in pH. Hence, to understand the basicity of the compounds and their response to acid, the p*K*_a_ values of these synthesized compounds 6a–6c were measured. A series of pH titrations were carried out in an acetate buffer within the pH range of 2–9. The p*K*_a_ value corresponds to the pH value at half the maximum absorbance (*λ*_max_). The observed p*K*_a_ values from UV titration were found to be 3.32, 4.48, and 4.65 for compounds 6a, 6b, and 6c, respectively (Fig. S3†). The higher p*K*_a_ value observed for compound 6c indicates its more basic nature, attributed to its greater planarity (Fig. 5b) leading to intramolecular hydrogen bonding.^36^ It was observed that as the pH value decreases from 9 to 6, the absorbance of 6c at 450 nm gradually diminishes. At the same time, a new peak at 505 nm begins to emerge as the pH is further reduced from 5 to 2 with the appearance of an isosbestic point at 470 nm, which describes the equilibrium between the protonated 6c+2H^+^ and deprotonated form of 6c (Fig. 5a). Thus, such a significant bathochromic shift of 55 nm in absorption spectra from pH 6 to pH 5 switched into the chromogenic change for the pH sensor. The absorption intensity ratios of the two different species (*λ*^Abs^_505_ nm/*λ*^Abs^_450_ nm) were determined and plotted against pH values, as shown in Fig. 5b. From this figure, it is evident that there is a significant change in the intensity ratio within the pH range of 6.0 to 4.0. The obvious bathochromic deviation of electronic absorption transition corresponds to the charge transfer (CT) nature of compound 6c upon protonation. Also, it was noted that emission spectra experienced a hypsochromic shift along with the bathochromic shift from 565 nm to 590 nm with the decrease of pH from pH 9 to pH 2 (Fig. 5c). It was also observed that with a decrease in the pH, the absorbance of the compound increased at 505 nm, but under the same conditions, fluorescence was quenched. A possible reason can be the dominance of the non-radiative pathway over the radiative process. Selective protonation of the imine bond can be evident from increased stretching frequency in ATR-FTIR from 1586 cm^−1^ to 1646 cm^−1^ upon exposure to acidic pH (Fig. S1†).

The reversibility of protonation-induced spectral changes of 6c was investigated through a “protonation–deprotonation” approach (Fig. 5d). The absorption spectral feature at 505 nm of the protonated form 6c+2H^+^ was restored to 450 nm for 6c with a red solution of 6c+2H^+^ turning to the orange colour of 6c immediately upon gradual addition of NaOH to protonated derivative 6c+2H^+^ (Fig. 5d). However, the subsequent addition of HCl to the orange solution of 6c resulted in the re-appearance of enhanced absorption (*λ*_505_) spectral transitions along with red fluorescence colour (UV light) of the solution due to the formation of the protonated form 6c+2H^+^. The “protonation–deprotonation–protonation” processes in 6c were achieved by the addition of HCl followed by NaOH solution in a controlled manner (Fig. 5d). Such restoration of the photophysical properties in 6c was observed in an ‘on–off–on’ pattern with the sequential addition of HCl followed by NaOH, achieving this for at least seven cycles in one stretch (Fig. 5d). After that, a gradual decrease in absorbance was observed, which can be attributed to the formation of the salt from the acid–base reaction of HCl + NaOH. However, timely workup after a certain number of cycles makes it reversible for multiple times. The reversibility in photophysical signalling thus highlights its reusability, stability of the molecule, and potential utility as a pH sensor for practical application-based explorations. Moreover, these interesting phenomena are observed for all kinds of strong to weak organic and inorganic acids, such as acetic acid, trifluoroacetic acid, picric acid, hydrochloric acid, and sulfuric acid (Fig. S4†). To better appreciate the potential sensitivity to acid and base, we calculate *K*_sv_ for TFA and TEA to be 4.8 × 10^4^ and 3.0 × 10^4^, respectively. This shows the sensitivity of 6c and 6c+2H^+^ towards TFA and TEA (Fig. S5–S8†).

**Fig. 6:**
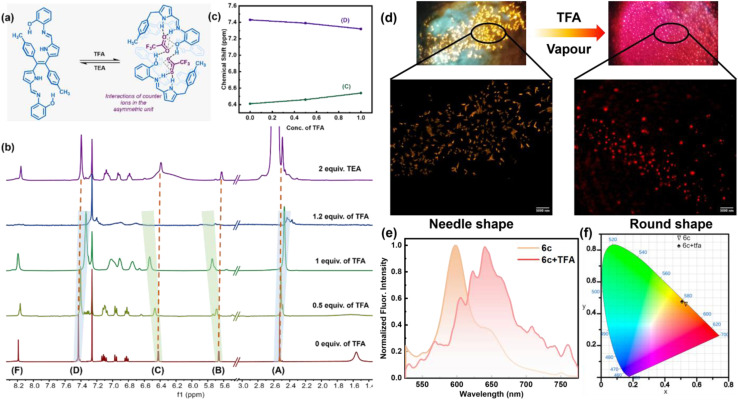
(a) Supramolecular switch of 6c with the addition of TFA and TEA. (b) Partial ^1^H NMR spectra of 6c upon gradual addition of TFA (CDCl_3_, r.t., 500 MHz) and reversible with addition of TEA. (c) Fitting of the observed variation in the chemical shifts of protons H_c_ and H_d_ plotted against the total concentration of added guest (TFA). (d) Confocal microscopy image showing needle shape crystals for compound 6c and spherical shape crystals when exposed to TFA vapour (6c+TFA), with excitation at 406 nm. (e) Solid state emission profile for 6c and 6c+TFA. (f) CIE diagram of 6c (CIE coordinates 0.531, 0.457) and 6c+TFA (CIE coordinates: 0.508, 0.476).

Consequently, to understand the change of molecular properties of chromophore 6c under an acidic medium followed by a basic medium, an ^1^H NMR titration experiment was performed in CDCl_3_ on ligand 6c upon the addition of TFA in a controlled manner, followed by TEA (Fig. 6b). It was observed that with the gradual addition of TFA, the –OH peak at 1.56 ppm vanished, and two sets of β-protons of two pyrroles were slightly down fielded with a broadening of spectra from 5.66 to 5.78 ppm and 6.41 ppm to 6.54 ppm respectively which indicates the interactions of the pyrrolic N–H protons with the corresponding acetate anion. However, both the tolyl ring and the –CH_3_ group in the tolyl groups also broaden with a slightly up-field shift from 7.42 ppm to 7.33 ppm and 2.52 ppm to 2.42 ppm. Interestingly, the above two continuous chemical-shifts provides a mirror image-like plot when the conc. of TFA is plotted against δ-ppm (Fig. 6c). This indicates the possibility of structural reorientation by diminishing the other noncovalent interactions. Similarly, other aromatic peaks, along with all other peaks, broadened significantly, representing the equilibrium between the 6c ligand and the complex 6c+2H^+^ (Fig. 6a). The complete disappearance of all the NMR signals upon addition of 1.2 equiv. of TFA indicates the complete formation of complex 6c+2H^+^. This indicates the possibility of a 1 : 1 adduct formation, which can be supported by Job's plot according to the method of continuous variations (Fig. S9†). Interestingly, with the addition of two equivalents of TEA, all the NMR peaks corresponding to 6c reappeared at their original positions, providing clear evidence of the breaking of the complex 6c+2H^+^ and reversibility of the supramolecular assembly (Fig. 6b). Furthermore, we visualized a significant morphological change in single crystal 6c when exposed to the TFA vapour under a confocal microscope. Initially, 6c exhibits needle-shaped crystals. However, when exposed to the TFA vapour, it was transformed into irregular-spherical shape morphology. This clearly indicates that there is a possibility of an interesting crystal-to-crystal transformation (Fig. 6d). The transition of crystalline morphology was further observed in the solid-state colourimetric change from orange to red with a solid-state fluorescence bathochromic shift of 44 nm (Fig. 6e and f).

**Fig. 7:**
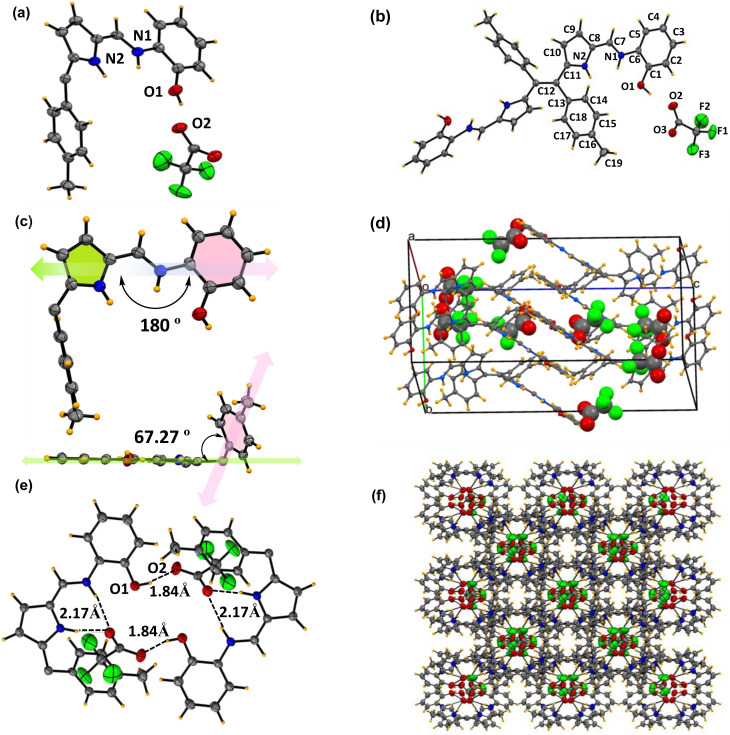
X-ray crystal structures of complex 6c+2H^+^. (a) Asymmetric unit (thermal ellipsoid are drawn with 50% probability). (b) Building block unit of complex 6c+2H^+^. (c) Inter-planar angle between pyrrole, toluene, and aminophenol from the top and side views in the asymmetric unit. (d) The number of trifluoroacetate anions present per unit cell structure. (e) Host guest supramolecular assembly between the trifluoro acetate anion and Schiff base through inter-molecular hydrogen bonding. (f) 3-Dimensional supramolecular assembly between the acetate anion and Schiff-base (CCDC No. of 6c+2H^+^2348084).

To confirm the formation of complex 6c+2H^+^ under acidic pH, we grew single crystals from a 1 : 1 mixture of Schiff base 6c and TFA in a methanol–chloroform (1 : 1) solvent system. The X-ray structure of complex 6c+2H^+^ is represented in Fig. 7. Complex 6c+2H^+^ crystallized in the monoclinic crystal system with the *C*2/*c* space group having lattice parameters: *a* = 13.3398(8) Å, *b* = 12.3038(7) Å, *c* = 25.4069(13) Å, and unit cell volume of 4133.33(4) Å. ORTEP drawing of the Schiff-base with 50% thermal ellipsoids shown in Fig. S10–S12.† All the crystallographic parameters, including relevant bond angles and bond distances, are tabulated in Tables S2–S8.†

The asymmetric unit of the complex 6c+2H^+^ shows that half of the ligand unit, along with one trifluoro acetate ion (CF_3_COO^−^) moiety (Fig. 7a), is held together by strong inter-molecular hydrogen bonding between O2 and H1 with a bond distance of 1.83(1) Å as shown in Fig. 7e. Like compound 6c, complex 6c+2H^+^ also contains one imine bond with a slight increment in bond distance to 1.30(4) Å. Moreover, the tolyl group deviates at an angle of 70.14° with respect to the rest of the Schiff base moiety plane (Fig. 7c). Notably, there is no such π⋯π interaction in the crystal lattice of 6c+2H^+^ like 6c. However, it forms a very stable supramolecular assembly through non-covalent interaction between the protonated ligand and CF_3_COO^−^, which orients itself in such a way that the CF_3_COO^−^ occupies the intermolecular void space in the crystal lattice (Fig. 7d and f). In the unit cell of complex 6c+2H^+^, eight ligands interact with eight CF_3_COO^−^ and hold together *via* strong intermolecular hydrogen bonding between the –NH protons of both pyrrole and imine units complemented by the OH proton of the phenol moiety with CF_3_COO^−^ with a bond distance of 1.83(8) Å, 2.18(8) Å and 1.85(1) Å respectively as shown in Fig. 6e. This provides clear evidence of the 1 : 1 adduct between the Schiff base 6c and CF_3_COO^−^ to form complex 6c+2H^+^ involving supramolecular host–guest interactions where the protonated Schiff base acts as the host and CF_3_COO^−^ acts as the guest, as illustrated in Fig. 6d. Moreover, the reversible colourimetric switch due to this host–guest interaction was observed in the solid state of 6c in the presence of TFA vapour to form 6c+2H^+^ and has been validated by PXRD (Fig. S13†), which indicates the possibility of crystal-to-crystal transfer in the presence of an acidic medium.

Comparing both of the X-ray structures, compound 6c shows high planarity with π⋯π stacking and hydrogen bonding interactions between two different units and growing in 1D to form a layer structure. However, it is interesting to note that in complex 6c+2H^+^, the π⋯π interactions were lost, and new host–guest interactions were observed, resulting in a highly stable supramolecular assembly. Due to these multidirectional supramolecular host–guest interactions, the highly planar ligand 6c transforms into a pseudo-polymeric 3D geometry, where all of the CF_3_COO^−^ occupies the void space of the crystal lattice and holds the Schiff-base through strong inter-molecular hydrogen bonding.

**Fig. 8:**
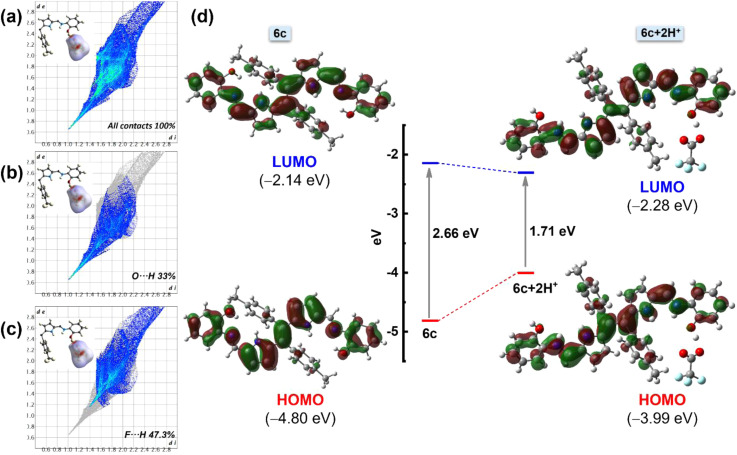
Theoretical analysis of 6c and 6c+2H^+^. Hirshfeld surface (mapped over *d*_norm_) analysis and corresponding overall fingerprints for (a) the asymmetric unit of complex 6c+2H^+^ with all possible interactions. (b) O⋯H interactions and (c) F⋯H interactions showing the individual contribution of each interaction to the total Hirshfeld surface of complex 6c+2H^+^. (d) Selected frontier molecular orbital with energy level diagrams of compounds 6c and 6c+2H^+^.

To understand the interactions and the connectivity present in the complex 6c+2H^+^, Hirshfeld surface analysis was further performed with CrystalExplorer17.5. In the 3D surface diagram, the red spot represents the presence of strong hydrogen bonds (H⋯O), which bring the participating atoms within a distance shorter than the sum of van der Waals radii. From Fig. 8b, the O⋯H interactions are the shortest, with a de + di distance of ∼1.70 Å and a 33% contribution to the total Hirshfeld surface. On the other hand, F⋯H interactions contribute to the Hirshfeld surface at 47.3% (Fig. 8c). Additionally, there are minimal contributions of other interactions like N⋯H interactions and C⋯H interactions, *etc.*, to quantify 100% contacts (Fig. 8a). This result implies that the presence of O⋯H and F⋯H interactions plays a major role in the formation of this kind of three-dimensional supramolecular assembly, 6c+2H^+^, which is consistent with the results of the single-crystal structure analysis.

Furthermore, a DFT study was performed to gain more insight into the structural, optical, and electronic properties of molecules 6a–6c and the protonated species 6c+2H^+^. Density functional theory (DFT) calculations were performed using the B3LYP/6-31 g(d,p) basic set. The stable, optimized structure of compounds 6a–6c at their ground state (S_0_) with selected geometrical parameters is presented in Fig. S14.† The optimized structures of 6b and 6c are identical to the obtained crystal structures. The selected frontier molecular orbital (FMO) analysis and the calculation results revealed that the electronic density of the highest occupied molecular orbital (HOMO) of ligands 6a, 6b, and 6c was delocalized over the entire molecule except the tolyl moiety. The lowest occupied molecular orbital (LUMO) of the molecules also shows a similar electron density distribution with a slight shifting of electron density to the tolyl core (Fig. S15†). Particularly, in the case of 6c, the HOMO–LUMO energy gap was calculated to be 2.66 eV. Interestingly, upon protonation, the energy gap became less and was calculated to be 1.71 eV, which can be attributed to the destabilization of the HOMO and the stabilization of the LUMO of chromophore 6c (Fig. 8d). This lowering of the HOMO–LUMO gap upon protonation is responsible for the redshift. Notably, lowering the energy gap is restricted not only through the influence of TFA but also by a similar energy profile diagram obtained for other acids (like HCl, HNO_3_, *etc.*) (Fig. S16†). Additionally, the TD-DFT studies of compounds 6a–6c show a good agreement with the experimental spectra (Fig. S17A†). The analysis indicates that the experimental UV-Vis absorption bands at 438 nm (6a), 455 nm (6b), and 465 nm (6c) have primarily resulted from HOMO to LUMO transitions. Other notable absorption bands, such as those at 358 nm (6a), 384 nm (6b), and 327 nm (6c), are largely attributed to transitions from the HOMO to LUMO+1. To analyze the nature of the absorption bands, we performed Natural Transition Orbital (NTO) analysis based on the calculated transition density matrices. This approach provides a compact representation of the transition density between the ground and excited states by expanding it into single-particle transitions, referred to as hole and electron states for a given excitation. In this context, we designate the unoccupied NTOs as “electron” orbitals and the occupied NTOs as “hole” orbitals. It is important to note that NTOs differ from the virtual and occupied molecular orbital (MO) pairs obtained from ground-state calculations.^37^ Fig. S17B† illustrates the NTOs for compounds 6a, 6b, and 6c. Based on our TD-DFT NTO analysis, the absorption bands observed in the 438–465 nm region for all the compounds can be characterized as optical excitations arising from transitions between the hole and electron orbitals in excited state 1. The hole NTOs are primarily localized on the dipyrroethene Schiff base conjugates, while the electron NTOs are distributed over both the dipyrroethene Schiff base conjugates and the *meso*-tolyl units in all Schiff base derivatives 6a, 6b, and 6c. This spatial distribution pattern is consistent for all derivatives (Fig. S17B†).

## 3. Conclusion and outlook

In summary, we have successfully demonstrated a synthetic strategy to access a novel class of pH-responsive, dual-state emissive, high fluorescent pyrrole-based chromophores by diformylation of DPE followed by condensation with aniline derivatives. This approach not only gives access to highly stable molecules with a large Stokes shift and good fluorescence quantum yield but also provides a great platform for various supramolecular interactions. Interestingly, these sets of molecules showcase pH-responsive crystal-to-crystal transfer phenomena through host–guest interactions by a combination of π⋯π and hydrogen bonding interactions, which is reversible for multiple-fold cycles. This nature has been established through various photophysical studies, NMR studies, and, most importantly, X-ray crystallographic analysis. Moreover, the reported results pave the way for the easy design and synthesis of finely tuned dipyrroethene-based chromophores, which hold potential in the rational design of highly fluorescent materials, including degradable and pH-responsive self-healing polymers. Moreover, this study opens new avenues for exploring innovative concepts in dynamic supramolecular chemistry, showcasing the versatility and potential applications in various scientific and industrial fields, including sensing, material applications and bioimaging.

## Data availability

The data supporting this article has been included as part of the ESI.† CCDC No. of 6b2368499; CCDC No. of 6c2345700; CCDC No. of 6c+2H^+^2348084.

## Author contributions

DM and MR: conceptualization of the work. DM: synthesis, characterization, data curation and writing-original manuscript draft. AS: crystallography and correction of the manuscript. KCB: writing-photophysical part, NTO analysis and correction of the manuscript. MR: supervision, validation and writing, reviewing and editing. AS and KCB contributed equally.

## Conflicts of interest

There are no conflicts to declare.

## Supplementary Material

SC-OLF-D4SC07016J-s001

SC-OLF-D4SC07016J-s002
