# Supplementary material for: pH-responsive supramolecular switch of a rationally designed dipyrroethene-based chromophore
Source: Chem Sci. 2024 Dec 16;16(4):1772–82. doi: 10.1039/d4sc07016j (PMC11664251; doi:10.1039/d4sc07016j)
Supplement: SC-016-D4SC07016J-s001 [file SC-016-D4SC07016J-s001.pdf]

Supplementary Information (SI) for Chemical Science.
This journal is © The Royal Society of Chemistry 2024

Supporting Information

for

pH-Responsive Supramolecular Switch of Rationally Designed Dipyrroethene-Based Chromospheres

Debasish Mandal, Abani Sarkar,+ Kanhu Charan Behera+ and Mangalampalli Ravikanth*

+These two authors contributed equally

Indian Institute of Technology, Powai, Mumbai, 400076, India.
E-mail: ravikanth@chem.iitb.ac.in; Fax: +91-22-5723480; Tel: +91-22-5767176

Sl. No.	Contents	Page No.
1.	Experimental: Materials and methods	S1-S3
2.	Synthetic Procedure of all the compound **6a**, **6b**, **6c** and **6c+2H+**	S3-S5
3.	Optimization table for the synthesis of *α*, *α*'-diformyl DPE.	S5
4.	ATR-FTIR of compound **6c** and **6c+2H+**	S6
4.	Solid state absorption emission profile of **6c**	S6
5.	pKa determination of **6a-6c** from the plot of A_{max} vs pH plot	S7
6.	Protonation of compound **6c** in the presence of different acids	S7
7.	Quenching of luminescence property in the presence of TFA	S8
8.	Enhancement of luminescence property in the presence of TEA	S9
9.	Job's plot	S10
10.	Crystallographic parameters	S11-S14
11.	ORTEP Diagram	S15
12.	PXRD plot	S16
13.	DFT, TD-DFT optimized structure, HOMO-LUMO energy profile and NTO analysis.	S17-S18
14.	Characterization data of compounds **5**, **6a**, **6b** and **6c**: ^{1}H, ^{13}C, COSY and HRMS	S19-S25
15.	DFT-optimized XYZ Coordinates	S26-S35
16.	References	S35

MATERIALS AND METHODS

Materials:

The chemicals including pyrrole, trifluoroacetic acid (TFA), Triethyl amine (TEA) benzoyl chloride, etc. were procured from reputable suppliers such as Merck and TCI. Unless otherwise indicated, all additional chemicals utilized in the synthesis were of reagent-grade quality. Zinc dust was activated using HCl and water before use. All the solvents are purchased from commercially available sources and performed proper distillation before use. Column chromatography was conducted using silica gel (100-200 mesh) and basic alumina, ensuring the purity and separation of compounds during the purification process.

Methods:

- **NMR Spectroscopy:** All the ^1H and ^{13}C NMR spectra were recorded in $CDCl_3$ on Bruker 400 and 500 MHz instruments at room temperature. The frequencies for the ^{13}C nucleus are 100.06 and 125.77 MHz for 400 and 500 MHz instruments, respectively. Chemical shifts (δ) are stated in parts per million (ppm) using the residual $CHCl_3$ (7.26 ppm for 1H and 77.16 ppm for ^{13}C). Coupling constants J are given in Hz. Multiplicities are described as singlet (s), broad signal (br), doublet (d), triplet (t), quartets (q), quintet (quint), and multiplet (m).
- **Steady-state optical spectroscopy:** UV/Vis absorption spectra were recorded on the Shimadzu UV-Vis-NIR 3600 spectrophotometer or JASCO V-670 spectrophotometer. Fluorescence spectra were recorded on a Horiba Fluoromax-4 or Fluorolog-QM spectrofluorometer. All absorption and emission spectra were recorded in 10 mm pathlength quartz cuvettes.
- **HRMS** were recorded with Bruker Maxis impact mass spectrometer using ESI-TOF techniques in positive mode by dissolving the compound in either methanol or acetonitrile.
- **Fluorescence quantum yields** were determined [S1] in each case by comparing the corrected spectrum with that of Rhodamine 6G (Φ =0.95) in EtOH by taking the area under total emission using the procedure reported earlier. [S2] Excitation and emission slit width are kept at 5 nm, and

neutral filter 2 is used on the excitation side to keep counts in the desired range. The following formula was used to calculate the solution state quantum yield.

$$\Phi_S = \Phi_R \times \frac{I_S}{I_R} \times \frac{A_R}{A_S} \times \left(\frac{\eta_S}{\eta_R}\right)^2$$

Φ_S = Quantum yield of the sample.

Φ_R = Quantum yield of reference.

I_S = Integrated fluorescence area of the sample.

I_R = Integrated fluorescence area of the reference.

A_R = Absorbance of the reference at the excitation wavelength.

A_s = Absorbance of the sample at the excitation wavelength.

η_S = Refractive index of the sample.

η_R = Refractive index of the reference.

Radiative and non-radiative rate constant was calculated using the following formula.

$$k_r = \frac{\Phi_f}{\tau} \qquad k_{nr} = \frac{(1 - \Phi_f)}{\tau}$$

k_r = Radiative rate constant

k_{nr} = non-radiative rate constant.

τ = Fluorescence lifetime

Φ_f = Fluorescence quantum yield.

- **The exponential decay** curve of compound **6a**, **6b** and **6c** were fitted appropriately with a mono/biexponential equation. The average lifetime (τ_{av}) was calculated following the equations depicted in the literature.[S3]
- **X-Ray Analysis**: Single yellow needle-shaped crystals of **6b** and **6c** were used as supplied. A suitable crystal with dimensions 0.10 × 0.20 × 0.30 mm^3 was selected and mounted on a Bruker APEX-II CCD diffractometer. The crystal was kept at a steady T = 150.15 K during data collection. The structure was solved with the Olex 2. Solve 1.5 (Bourhis et al., 2015) solution program using dual space methods and by using Olex 2 1.5-dev (Dolomanov et al., 2009) as

the graphical interface. The model was refined with olex2. refine 1.5-dev (Bourhis et al., 2015) using full matrix least squares minimization on F2.[S4-S5]

- Quantum chemical calculations (gas phase/vacuum) for ground state energy minimized structures for **6c** and **7** were done employing density functional theory (DFT) in a Gaussian 09W program package.[S6] The ground state structural elucidation involved in optimization using DFT-based Beck-3 Lee Young Parr (B3LYP) functional where 6 311G basis sets were used.[S7]
- To generate the Hirshfeld surfaces, the crystallographic information file (.CIF) was imported into the CrystalExplorer17.5 Program.[7],[8] Three-dimensional (3D) Hirshfeld surface maps are generated with dnorm using a red-white-blue colour scheme, where red highlights shorter contacts, white is used for contacts around the rvdW separation, and blue is for longer contacts.

$$d_{norm} = \frac{d_i - r_i^{vdW}}{r_i^{vdW}} + \frac{d_e - r_e^{vdW}}{r_e^{vdW}}$$

Additionally, 2-D fingerprint plots, generated using external (d_e) and internal (d_i) distances, complement these 3-D surfaces, providing a quantitatively summary of the nature and the type of intermolecular interactions in the crystal.

SYNTHETIC PROCEDURE

General procedure for the synthesis of diformylation of dipyrroethenes: In a round bottom flask equipped with a condenser and nitrogen atmosphere, dimethylformamide (1.5 mmol, 5eq.) was taken and kept under ice-cold conditions. Under the nitrogen bubbling condition, phosphorus oxychloride (1.5 mmol, 5 eq.) was added and stirred for 10 minutes, which led to the formation of gel-like mixture. The reaction mixture was brought back to room temperature and reaction grade 1,2-dichloroethane (DCE, 5 ml) was added and stirred for 20 minutes at room temperature. Again, the reaction mixture was cooled to ice-cold conditions and *(E)*-dipyrroethene (0.3 mmol, 1 eq.) dissolved in 1,2-dichloroethane (5 mL) was added dropwise using a dropping funnel under an N_2 atmosphere. After the addition was over, the reaction mixture was refluxed at 80 °C for 6 hours. The reaction mixture was brought to room temperature, quenched with sodium bicarbonate and a

work-up was done using dichloromethane (CH_2Cl_2)-water (50:50). The crude reaction mixture was dried over anhydrous Na_2SO_4. The solvent was removed on a rotary evaporator under a high vacuum, and the resulting crude product was purified by silica-gel column chromatography using CH_2Cl_2 solvent. The solvent was removed to afford the two desired diformylated products in overall 82% yields.

(E)-diformyl-dipyrroethenes **5**. mp>350 °C; ^1H NMR (400 MHz, $CDCl_3$) δ 2.47 (s, 6H), 5.51 (dd, J = 2.6, 4.1 Hz, 2H), 6.64 (dd, J = 2.4, 4.2 Hz, 2H), 7.23 (d, J = 8.1 Hz, 4H), 7.31 (d, J = 7.8 Hz, 4H), 8.26 (s, 2H), 9.28 (s, 2H). ^{13}C {^1H} NMR (101 MHz, $CDCl_3$) δ 178.33, 139.77, 139.27, 136.07, 132.31, 131.12, 130.45, 129.88, 120.54, 115.30, 21.44. HR-MS (ESI-TOF) *m/z*: $[M + H]^+$ calcd. for $C_{26}H_{22}N_2O_2$, 395.1750; found, 395.1750.

General procedure for the synthesis of compounds **6a**, **6b,** and **6c**: Diformyl-dipyrroethene (0.77mmol, 1eq.) and Aniline or *ortho*-substituted aniline (1.92 mmol, 2.5 eq.) was taken in a round-bottom flask and dissolved in reaction grade methanol. A catalytic amount of glacial acetic acid was added and the reaction mixture was refluxed at 65 °C for six hours under a nitrogen atmosphere. The reaction led to the formation of orange precipitation in the reaction mixture. The mixture was brought to ice-cold condition and kept for 30 min. Then the reaction mixture was filtered through the Whatman grade filter paper. The residue was washed multiple times in cold methanol to obtain the pure Schiff base as a solid orange or light orange compound **6a**, **6b**, and **6c** in selectively *(E)*-configuration.

6a: (Yield: 62%); mp>350 °C; ^1H NMR (500 MHz, $CDCl_3$) δ 2.47 (s, 6H), 5.57 (d, J = 3.9 Hz, 2H), 6.37 (d, J = 3.9 Hz, 2H), 7.05 (d, J = 7.7 Hz, 4H), 7.15 (t, J = 7.4 Hz, 2H), 7.30 (t, J = 7.4 Hz, 4H), 7.34 (d, J = 14.8 Hz, 8H), 8.01 (s, 2H). ^{13}C NMR {^1H} (126 MHz, $CDCl_3$) δ 29.59, 115.04, 115.95, 120.72, 125.19, 128.93, 129.94, 130.14, 130.84, 131.20, 135.70, 137.11, 138.36, 147.91, 151.46. HR-MS (ESI-TOF) *m/z*: $[M + H]^+$ calcd. for $C_{38}H_{32}N_4$, 545.2712; found, 545.2711.

6b: (Yield: 67%); mp>350 °C; ^1H NMR (400 MHz, $CDCl_3$) δ 2.48 (s, 6H), 5.59 (d, J = 4.0 Hz, 2H), 6.49 (d, J = 4.0 Hz, 2H), 7.01 (m, 4H), 7.35 (s, 8H), 7.62 (m, 2H), 8.39 (m, J = 0.9, 1.9, 4.9 Hz,

2H), 8.75 (s, 2H). ^{13}C NMR {^{1}H} (101 MHz, $CDCl_3$) δ 21.37, 114.92, 115.44, 115.46, 116.43, 118.20, 126.62, 127.85, 130.00, 130.13, 131.89, 136.79, 137.36, 137.40, 138.24, 141.73, 145.46. HR-MS (ESI-TOF) *m/z*: $[M + H]^+$ calcd. for $C_{38}H_{34}N_6$, 575.2929; found, 575.2929.

6c: (Yield: 73%); mp>350 °C; ^{1}H NMR (400 MHz, $CDCl_3$) δ 2.53 (s, 6H), 5.66 (d, *J* = 3.9 Hz, 2H), 6.42 (d, *J* = 3.9 Hz, 2H), 6.78 (m, 2H), 6.96 (dd, *J* = 1.4, 8.1 Hz, 2H), 7.06 (m, 4H), 7.43 (d, *J* = 1.0 Hz, 8H), 8.19 (s, 2H). ^{13}C NMR {^{1}H} (101 MHz, $CDCl_3$) δ 21.24, 114.35, 114.96, 116.14, 116.46, 119.75, 127.52, 129.94, 130.26, 130.63, 131.58, 135.29, 136.85, 138.10, 139.39, 144.73, 151.66. HR-MS (ESI-TOF) *m/z*: $[M + H]^+$ calcd. for $C_{38}H_{32}N_4O_2$, 577.2593; found, 577.2592

6c+2H$^+$: HR-MS (ESI-TOF) *m/z*: $[M]^+$ calcd. for $C_{42}H_{34}F_6N_4O_6$, 804.2293; found, 804.2292

SUPPORTING FIGURE AND TABLE

Table S1 Optimization table for the synthesis of *α*, *α*'-diformyl DPE (**5**).

S. N	*Equivalent of $POCl_3$*	*Equivalent of DMF*	*Temp (°C)*	*Time (h)*	*Yield*
1.	2	2	65	4	33%
2.	2	2	80	6	45%
**3.**	**5**	**5**	**80**	**6**	**82%**
4.	5	5	85	8	80%
5.	5	7	85	8	78%
6.	7	7	85	6	75%
7.	7	7	80	4	77%

**The reaction was carried out by following the mentioned protocol in the previous section. 1,2 DCE solvent was used for the mentioned reactions in all the time.*

IR-Plot

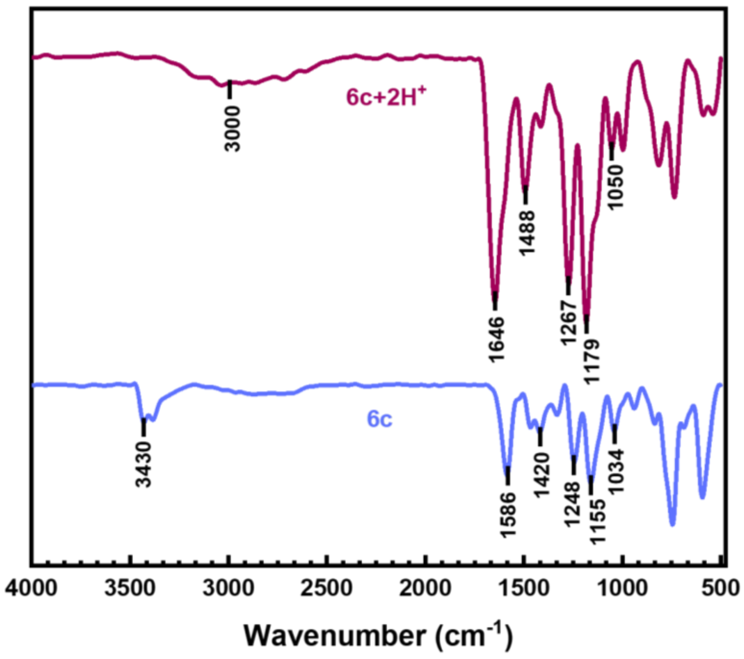

Figure S1 ATR-FTIR of compound **6c** and **6c**+2H^{+}

Excitation Spectra and Solid-State UV-Vis and emission Spectra

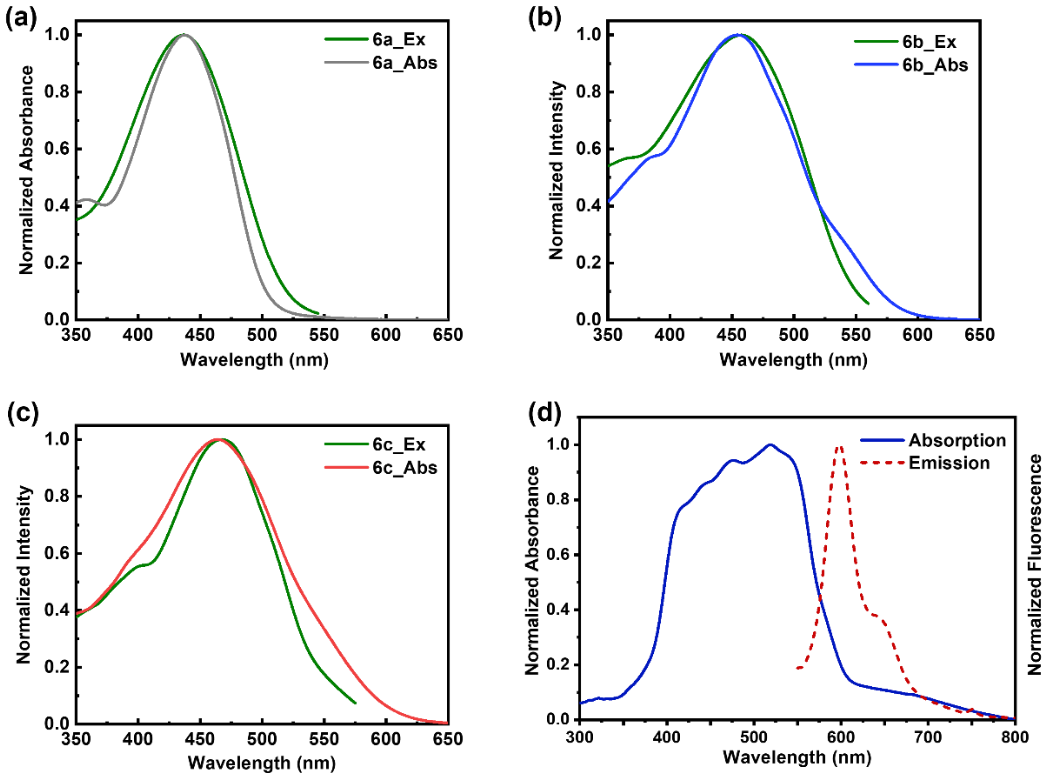

Figure S2 Excitation spectra of (a) **6a**, (b) **6b** and (c) **6c**, (d) solid state UV-Vis and emission spectra of **6c**

pKa Determination Plot

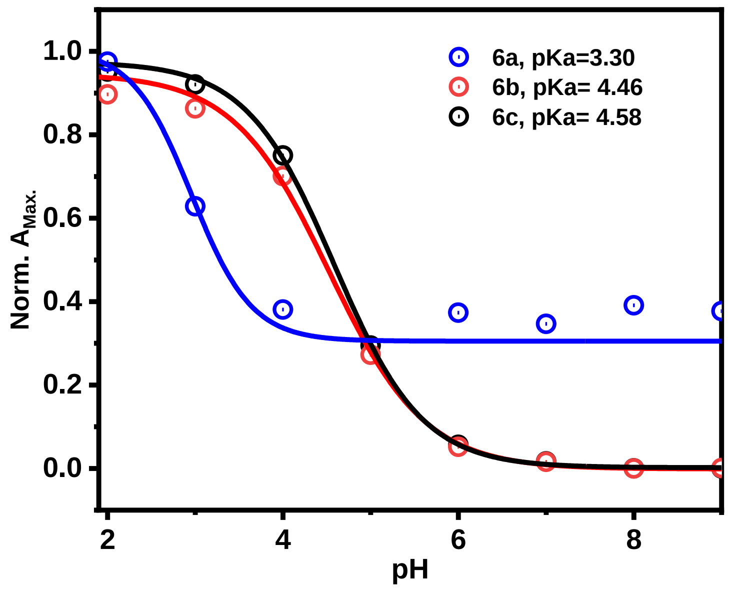

Figure S3 pKa determination of **6a-6c** from the plot of A_{max} vs pH plot

UV-Vis Spectra in Presence of Different Acid

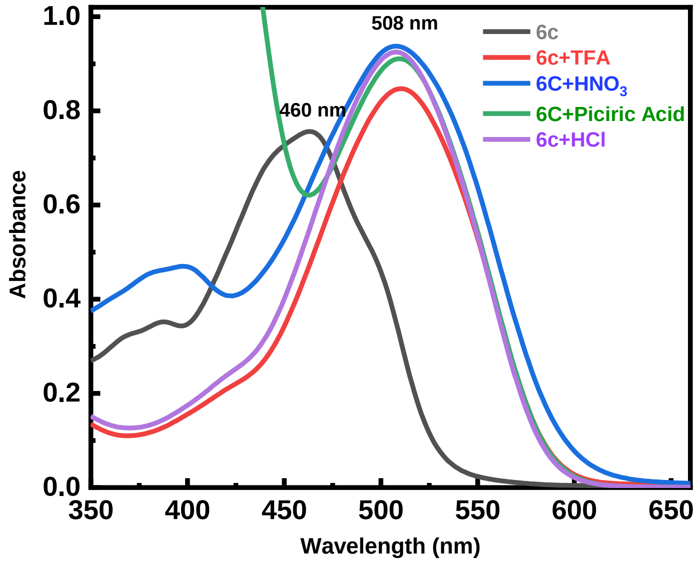

Figure S4 Protonation of compound **6c** in the presence of different acids.

Quenching of luminescence property in presence of TFA

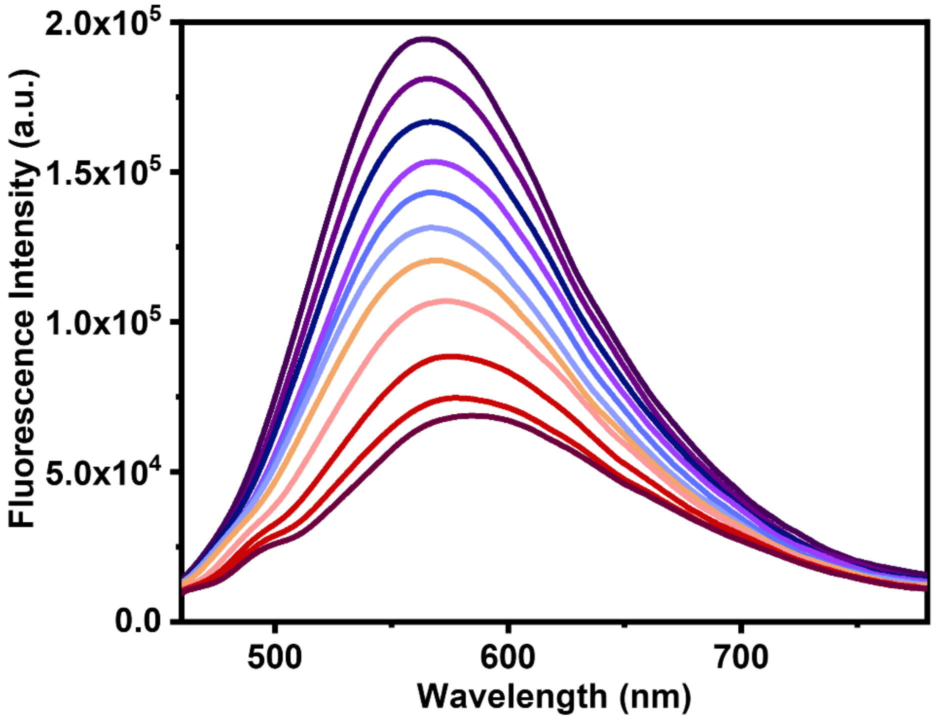

Figure S5 Fluorescence Quenching of molecules **6c** in the presence of TFA

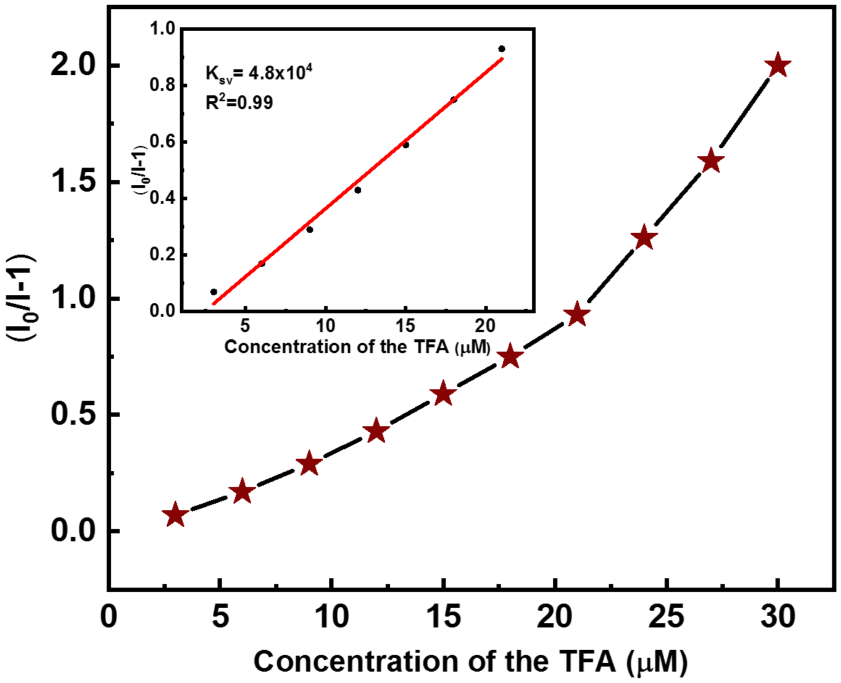

Figure S6 SV-Plot for addition of TFA compound **6c** (Inset: Linear fitting within lower concentration region)

Enhancement of luminescence property in presence of TEA

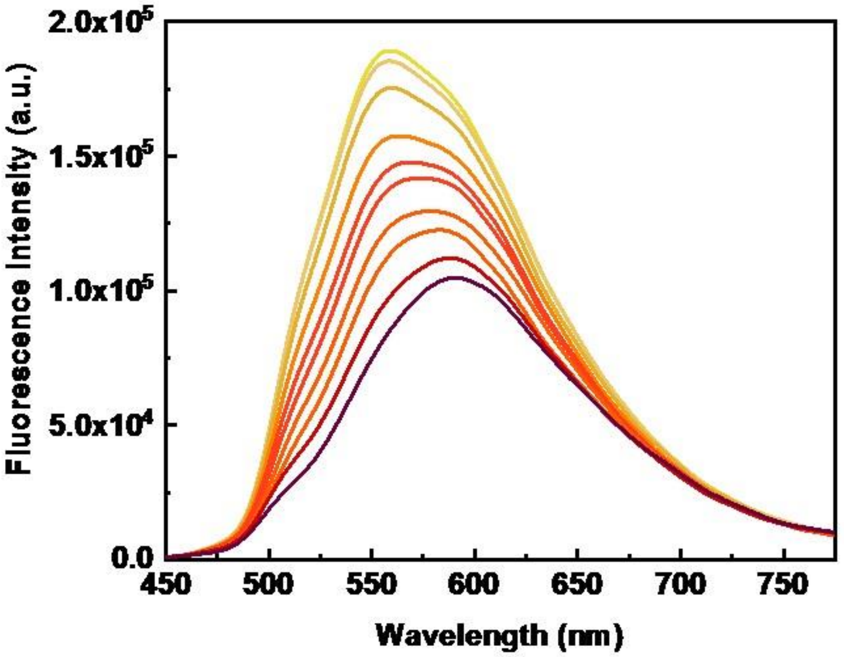

Figure S7 Fluorescence enhancement of molecules **6c+2H**$^+$ in the presence of TEA.

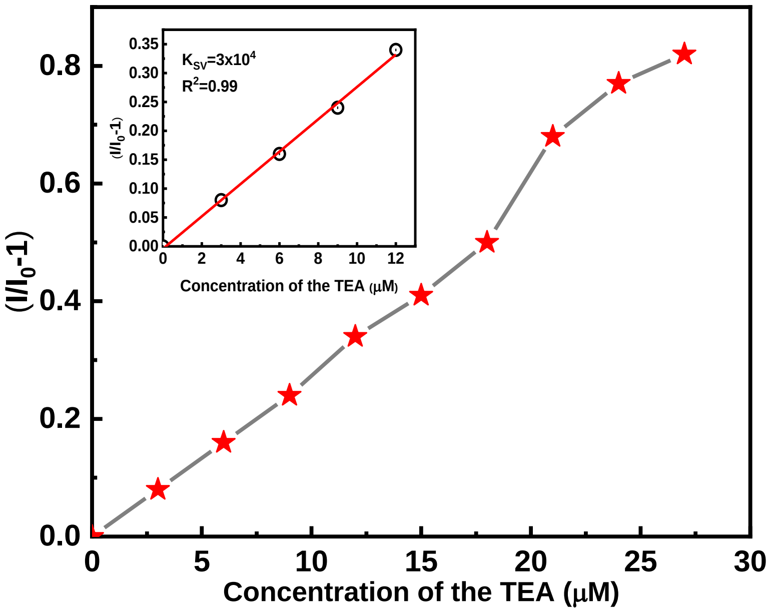

Figure S8 SV-Plot for the addition of TEA compound **6c+2H**$^+$ (Inset: Linear fitting within lower concentration region)

Job's plot

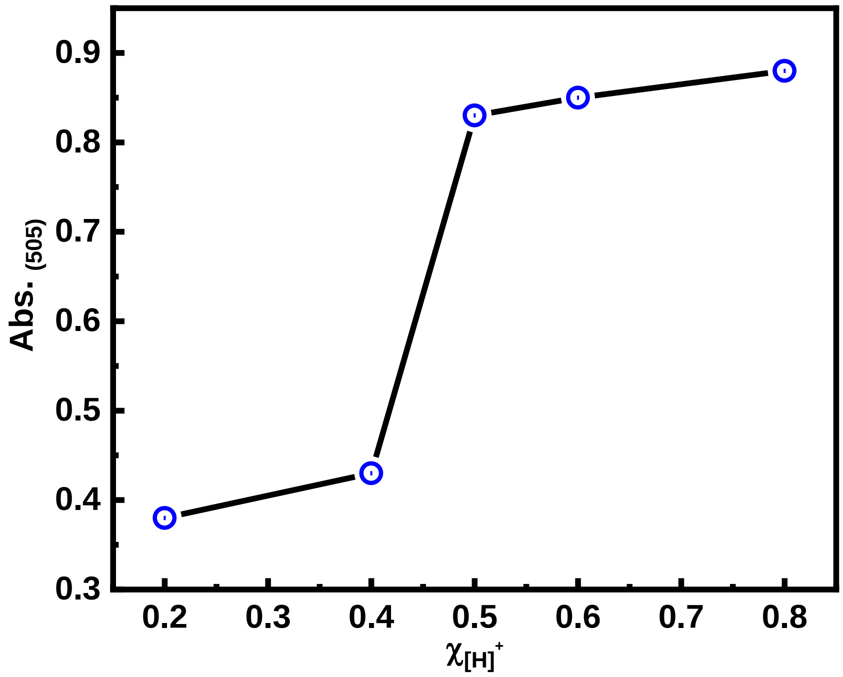

Figure S9 Job's plot according to the method of continuous variations, indicating the 1:1 stoichiometry for compound **6c+2H**$^+$

Crystal table

Table S2 Crystallographic data and processing parameters of compounds **6b**, **6c**, and **6c+2H$^+$**

Compounds	**6b**	**6c**	**6c+2H$^+$**
CCDC			
Empirical formula	$C_{19}H_{17}N_3$	C38H32N4O2	$C_{50}H_{38}F_6N_4O_6$
Formula weight	287.367	576.67	804.73
Temperature/k	105.00	150.15	150.15
Crystal system	triclinic	triclinic	monoclinic
Space group	P-1	P-1	C2/c
a/Å	9.295(2)	7.2089(7)	13.3398(8)
b/Å	9.499(2)	9.3281(9)	12.3038(7)
c/Å	10.348(2)	11.0857(13)	25.4069(13)
α/°	79.673(10)	97.148(9)	90
β/°	65.183(8)	92.220(9)	97.608(5)
γ/°	63.713(9)	96.254(8)	90
Volume/Å^3	743.5(3)	734.23(13)	4133.3(4)
Z	2	1	4
ρ_{calc}g/cm^3	1.284	1.304	1.293
μ/mm^{-1}	0.077	0.082	0.105
F(000)	304.2	304.0	1872.0
Crystal size/mm^3	0.2 × 0.01 × 0.01	0.25 × 0.2 × 0.1	2.1 ×0.2 × 0.2
Radiation	Mo Kα (λ = 0.71073)	Mo Kα (λ = 0.71073)	Mo Kα (λ = 0.71073)
2θ	4.34 to 50.34	3.708 to 50	4.94 to 50
Index ranges	-11 ≤ h ≤ 11, -11 ≤ k ≤ 11, -12 ≤ l ≤ 12	-8 ≤ h ≤ 8, -11 ≤ k ≤ 11, -13 ≤ l ≤ 13	-15 ≤ h ≤ 15, -14 ≤ k ≤ 14, -30 ≤ l ≤ 30
Reflections collected	19667	18304	31728
Independent reflections	2666 [Rint = 0.1581, Rsigma = 0.0960]	2537 [Rint = 0.1144, Rsigma = 0.0564]	3616 [Rint = 0.1083, Rsigma = 0.0665]
Data/restraints/ parameters	2666/0/219	2537/0/201	3616/0/264
Goodness-of-fit F^2	1.099	1.071	1.059
Final R indexes [i>=2σ (i)]	R1 = 0.0586, wR2 = 0.1376	R1 = 0.0874, wR2 = 0.2197	R1 = 0.0740, wR2 = 0.1819
Final R indexes [all data]	R1 = 0.1127, wR2 = 0.1736	R1 = 0.1033, wR2 = 0.2307	R1 = 0.1174, wR2 = 0.2075
Largest diff. peak/hole / e Å^{-3}	0.38/-0.39	0.41/-0.30	0.53/-0.37

Bond Angle-Bond length Table

Table S3 Bond Lengths for **6b**.

Atom	Atom	Length/Å	Atom	Atom	Length/Å
N_1	C_9	1.374(3)	C_6	C_7	1.387(4)
N_1	C_{12}	1.376(3)	C_{10}	C_{11}	1.397(4)
N_2	C_{13}	1.281(3)	C_{14}	C_{15}	1.419(4)
N_2	C_{14}	1.423(3)	C_{14}	C_{19}	1.389(4)
N_3	C_{15}	1.382(4)	C_3	C_4	1.376(4)
C_5	C_8	1.492(4)	C_3	C_2	1.392(4)
C_5	C_6	1.400(4)	C_7	C_2	1.388(4)
C_5	C_4	1.385(4)	C_2	C_1	1.496(4)
C_8	C_8^1	1.365(5)	C_{15}	C_{16}	1.390(4)
C_8	C_9	1.465(4)	C_{19}	C_{18}	1.371(4)
C_9	C_{10}	1.397(4)	C_{16}	C_{17}	1.372(4)
C_{12}	C_{13}	1.420(4)	C_{17}	C_{18}	1.388(4)
C_{12}	C_{11}	1.375(4)			

Table S4 Bond Angles for **6b**.

Atom	Atom	Atom	Angle/°	Atom	Atom	Atom	Angle/°
C_{12}	N_1	C_9	110.0(2)	C_{19}	C_{14}	N_2	123.6(3)
C_{14}	N_2	C_{13}	118.8(2)	C_{19}	C_{14}	C_{15}	118.9(3)
C_6	C_5	C_8	120.1(2)	C_{10}	C_{11}	C_{12}	107.8(3)
C_4	C_5	C_8	121.9(2)	C_2	C_3	C_4	121.3(3)
C_4	C_5	C_6	118.0(2)	C_3	C_4	C_5	121.5(3)
C_8^1	C_8	C_5	122.3(3)	C_2	C_7	C_6	122.0(3)
C_9	C_8	C_5	112.2(2)	C_7	C_2	C_3	117.3(3)
C_8	C_9	N_1	127.0(2)	C_1	C_2	C_3	121.1(3)
C_{10}	C_9	N_1	106.4(2)	C_1	C_2	C_7	121.6(3)
C_{10}	C_9	C_8	126.6(2)	C_{14}	C_{15}	N_3	119.2(3)
C_{13}	C_{12}	N_1	122.9(3)	C_{16}	C_{15}	N_3	122.7(3)
C_{11}	C_{12}	N_1	107.7(2)	C_{16}	C_{15}	C_{14}	118.0(3)
C_{11}	C_{12}	C_{13}	129.5(3)	C_{18}	C_{19}	C_{14}	122.0(3)
C_7	C_6	C_5	119.9(3)	C_{17}	C_{16}	C_{15}	121.7(3)
C_{11}	C_{10}	C_9	108.1(2)	C_{18}	C_{17}	C_{16}	120.3(3)
C_{12}	C_{13}	N_2	123.9(3)	C_{17}	C_{18}	C_{19}	119.0(3)
C_{15}	C_{14}	N_2	117.6(3)				

Table S5 Bond Lengths for **6c**.

Atom	Atom	Length/Å	Atom	Atom	Length/Å
O_1	C_{19}	1.378(5)	C_{11}	C_{12}	1.376(6)
N_1	C_9	1.375(5)	C_2	C_3	1.390(6)
N_1	C_{12}	1.378(5)	C_2	C_7	1.389(6)
N_2	C_{13}	1.274(5)	C_2	C_1	1.521(6)
N_2	C_{14}	1.425(5)	C_{13}	C_{12}	1.426(6)
C_9	C_8	1.460(5)	C_3	C_4	1.389(6)
C_9	C_{10}	1.396(5)	C_{14}	C_{19}	1.405(6)
C_8	C_8^1	1.370(7)	C_{14}	C_{15}	1.403(6)
C_8	C_5	1.498(5)	C_{19}	C_{18}	1.390(6)
C_5	C_6	1.397(5)	C_{15}	C_{16}	1.373(6)
C_5	C_4	1.389(5)	C_{18}	C_{17}	1.370(7)
C_{10}	C_{11}	1.409(6)	C_{17}	C_{16}	1.423(7)
C_6	C_7	1.391(6)			

Table S6 Bond Angles for **6c**.

Atom	Atom	Atom	Angle/°	Atom	Atom	Atom	Angle/°
C_9	N_1	C_{12}	110.1(3)	N_2	C_{13}	C_{12}	122.7(4)
C_{13}	N_2	C_{14}	121.5(4)	N_1	C_{12}	C_{13}	122.3(3)
N_1	C_9	C_8	126.7(3)	C_{11}	C_{12}	N_1	107.4(3)
N_1	C_9	C_{10}	106.9(3)	C_{11}	C_{12}	C_{13}	130.2(4)
C_{10}	C_9	C_8	126.4(3)	C_4	C_3	C_2	121.3(4)
C_9	C_8	C_5	111.7(3)	C_3	C_4	C_5	120.7(4)
C_8^1	C_8	C_9	126.0(4)	C_2	C_7	C_6	120.8(4)
C_8^1	C_8	C_5	122.3(4)	C_{19}	C_{14}	N_2	114.9(4)
C_6	C_5	C_8	121.6(3)	C_{15}	C_{14}	N_2	127.2(4)
C_4	C_5	C_8	120.2(3)	C_{15}	C_{14}	C_{19}	117.8(4)
C_4	C_5	C_6	118.1(3)	O_1	C_{19}	C_{14}	118.7(4)
C_9	C_{10}	C_{11}	107.5(3)	O_1	C_{19}	C_{18}	119.6(4)
C_7	C_6	C_5	120.9(4)	C_{18}	C_{19}	C_{14}	121.7(4)
C_{12}	C_{11}	C_{10}	108.2(4)	C_{16}	C_{15}	C_{14}	121.4(4)
C_3	C_2	C_1	120.8(4)	C_{17}	C_{18}	C_{19}	119.3(4)
C_7	C_2	C_3	118.2(4)	C_{18}	C_{17}	C_{16}	120.6(4)
C_7	C_2	C_1	121.0(4)	C_{15}	C_{16}	C_{17}	119.2(5)

Table S7 Bond Lengths for **6C**+2H^{+}.

Atom	Atom	Length/Å	Atom	Atom	Length/Å
O_1	C_1	1.371(4)	C_{11}	C_{10}	1.408(5)
O_3	C_{20}	1.247(5)	C_6	C_1	1.396(5)
O_2	C_{20}	1.229(5)	C_6	C_5	1.390(5)
N_2	C_8	1.378(4)	C_{12}	C_{12}	1.365(7)
N_2	C_{11}	1.350(4)	C_1	C_2	1.375(5)
N_1	C_7	1.310(5)	C_{18}	C_{17}	1.398(5)
N_1	C_6	1.425(4)	C_{10}	C_9	1.380(5)
F_3	C_{21}	1.319(5)	C_5	C_4	1.379(5)
F_2	C_{21}	1.333(5)	C_{14}	C_{15}	1.390(5)
F_1	C_{21}	1.302(5)	C_2	C_3	1.382(5)
C_{13}	C_{12}	1.493(5)	C_{17}	C_{16}	1.394(6)
C_{13}	C_{18}	1.391(5)	C_4	C_3	1.391(6)
C_{13}	C_{14}	1.394(5)	C_{16}	C_{15}	1.383(6)
C_7	C_8	1.394(5)	C_{16}	C_{19}	1.500(5)
C_8	C_9	1.400(5)	C_{20}	C_{21}	1.531(6)
C_{11}	C_{12}				

Table S8 Bond Angles for **6C**+2H^{+}.

Atom	Atom	Atom	Angle/°	Atom	Atom	Atom	Angle/°
C_{11}	N_2	C_8	109.6(3)	C_9	C_{10}	C_{11}	107.7(3)
C_7	N_1	C_6	126.3(3)	C_4	C_5	C_6	119.4(4)
C_{18}	C_{13}	C_{12}	121.0(3)	C_{15}	C_{14}	C_{13}	120.7(4)
C_{18}	C_{13}	C_{14}	118.5(3)	C_1	C_2	C_3	120.1(4)
C_{14}	C_{13}	C_{12}	120.6(3)	C_{16}	C_{17}	C_{18}	121.2(4)
N_1	C_7	C_8	126.5(3)	C_{10}	C_9	C_8	107.5(3)
N_2	C_8	C_7	125.7(3)	C_5	C_4	C_3	120.3(4)
N_2	C_8	C_9	107.4(3)	C_{17}	C_{16}	C_{19}	120.8(4)
C_7	C_8	C_9	126.9(3)	C_{15}	C_{16}	C_{17}	117.9(3)
N_2	C_{11}	C_{12}	121.4(3)	C_{15}	C_{16}	C_{19}	121.3(4)
N_2	C_{11}	C_{10}	107.7(3)	O_3	C_{20}	C_{21}	114.2(4)
C_{10}	C_{11}	C_{12}	130.8(3)	O_2	C_{20}	O_3	129.9(4)
C_1	C_6	N_1	116.3(3)	O_2	C_{20}	C_{21}	115.9(4)
C_5	C_6	N_1	123.5(3)	C_2	C_3	C_4	120.0(4)
C_5	C_6	C_1	120.2(3)	C_{16}	C_{15}	C_{14}	121.4(4)
C_{11}	C_{12}	C_{13}	115.9(3)	F_3	C_{21}	F_2	103.9(4)
C_{12}^{1}	C_{12}	C_{13}	122.2(4)	F_3	C_{21}	C_{20}	111.7(3)
C_{12}^{1}	C_{12}	C_{11}	121.9(4)	F_2	C_{21}	C_{20}	111.2(4)
O_1	C_1	C_6	117.0(3)	F_1	C_{21}	F_3	109.6(4)
O_1	C_1	C_2	123.1(4)	F_1	C_{21}	F_2	104.8(4)
C_2	C_1	C_6	119.9(4)	F_1	C_{21}	C_{20}	114.7(4)
C_{13}	C_{18}	C_{17}	120.2(4)				

<u>ORTEP Diagram</u>

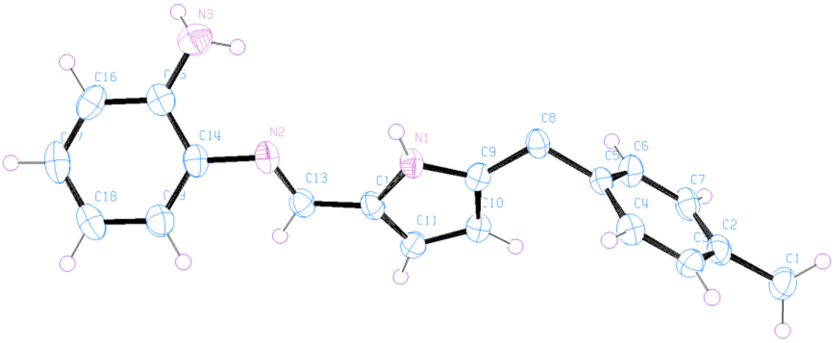

Figure S10 ORTEP drawing (ellipsoids at 50% probability) of complex **6b.**

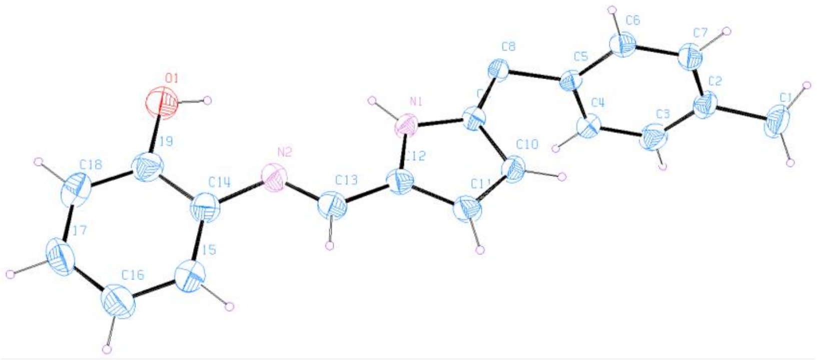

Figure S11 ORTEP drawing (ellipsoids at 50% probability) of complex **6c.**

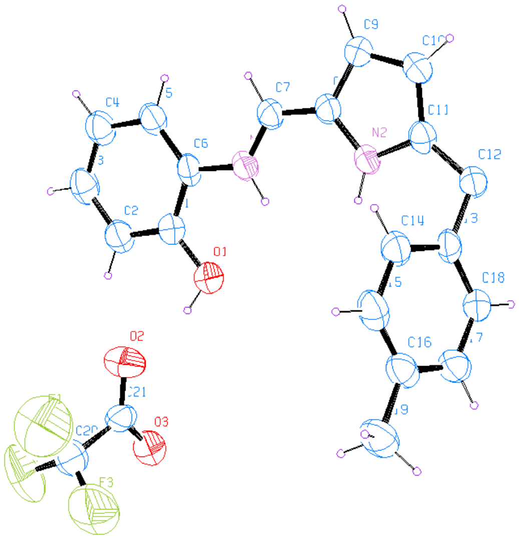

Figure S12 ORTEP drawing (ellipsoids at 50% probability) of complex **6c**+2H^{+}**.**

PXRD Plot

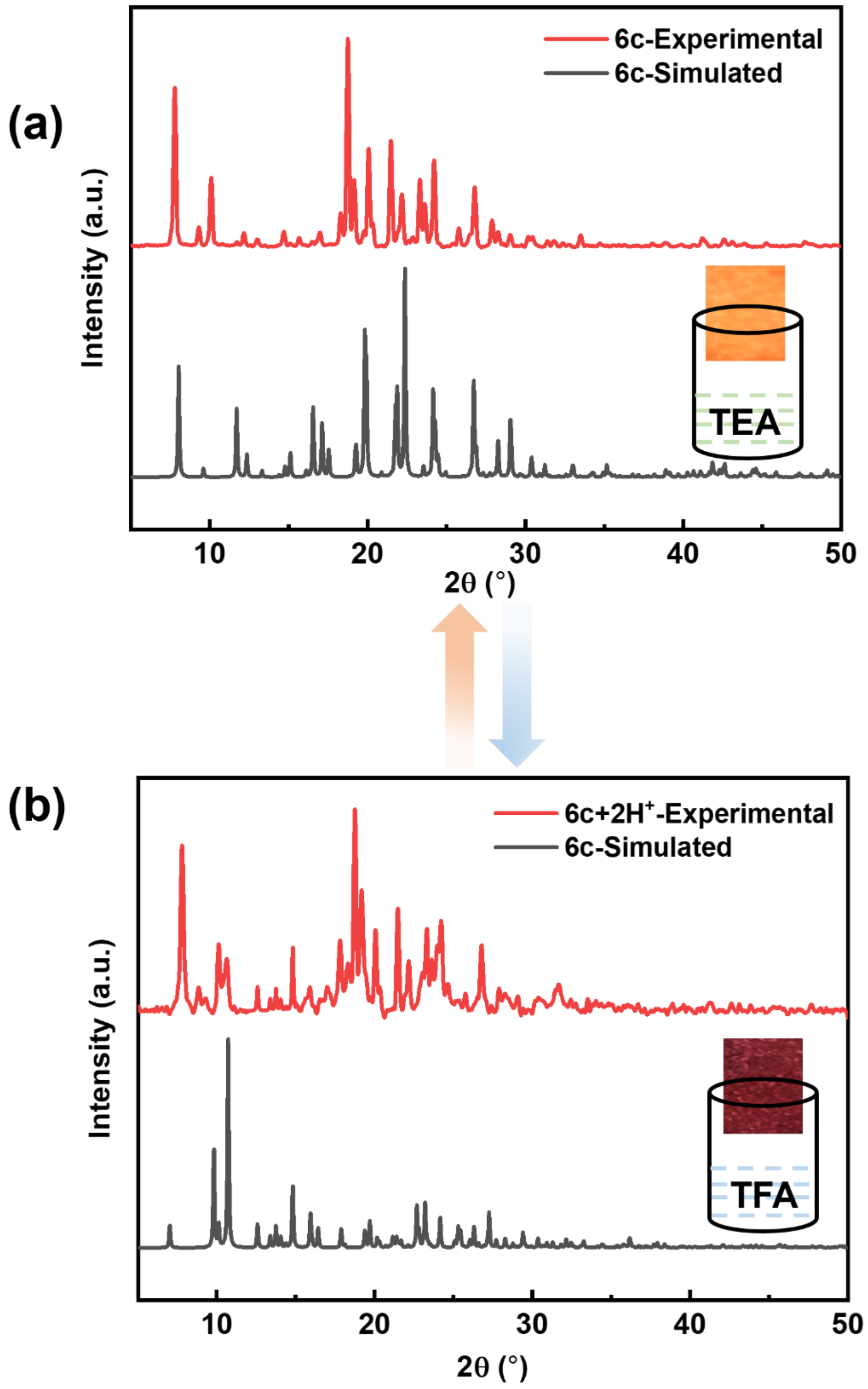

Figure S13 Simulated and experimental PXRD data of (a) **6c** under ambient conditions, (b) **6c** after being exposed to the TFA vapour, matching with the simulated data of **6c**+$2H^+$

DFT Optimized Structures

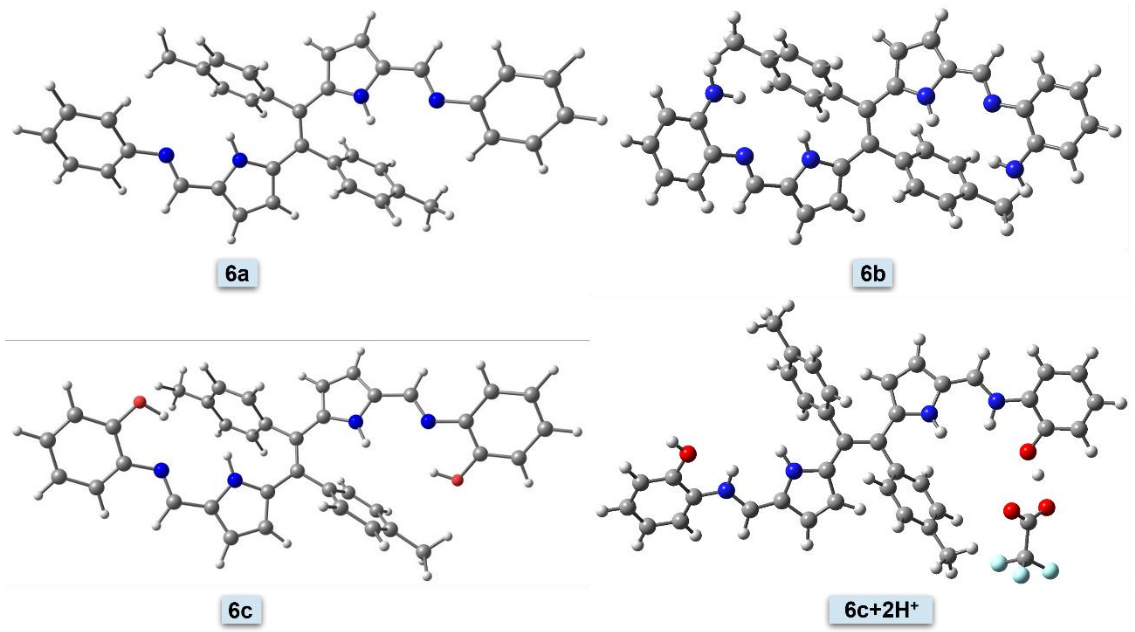

Figure S14 Optimized Structures of the ligand **6a**, **6b**, and **6c+2H$^+$**

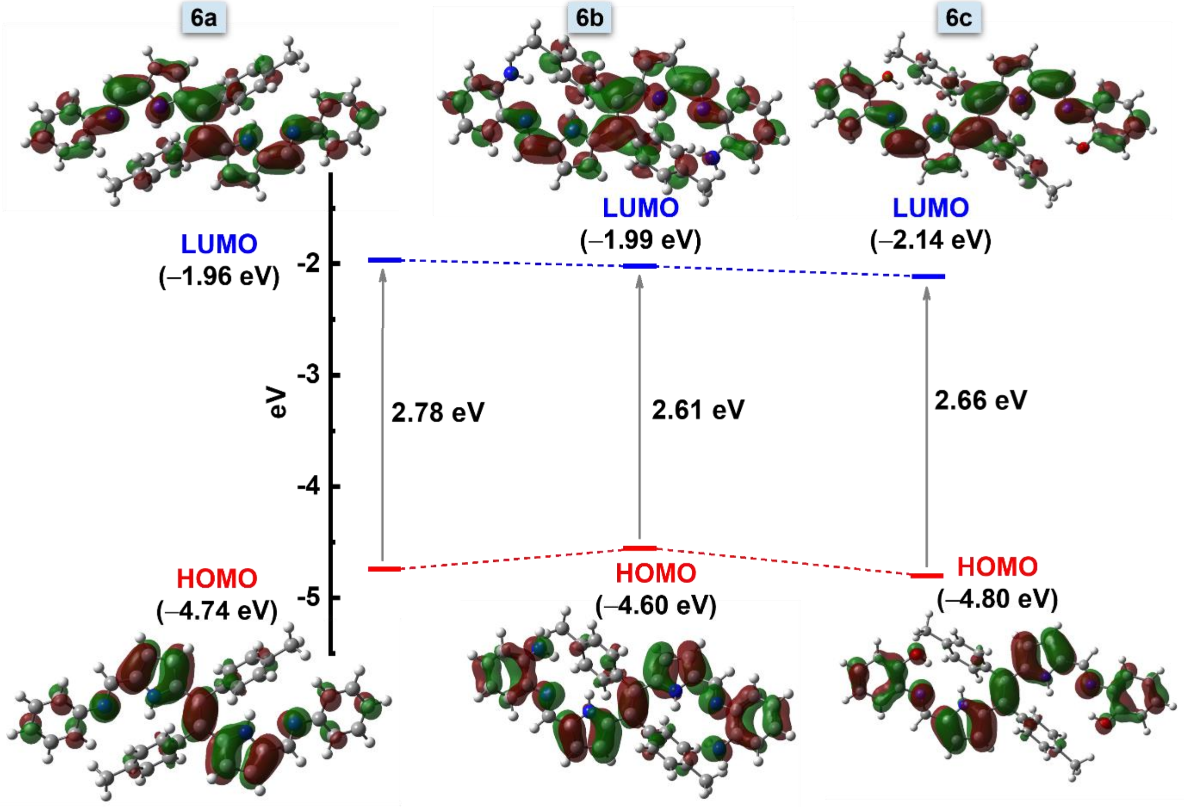

Figure S15 Energy profile diagram of the ligands **6a**, **6b,** and **6c.**

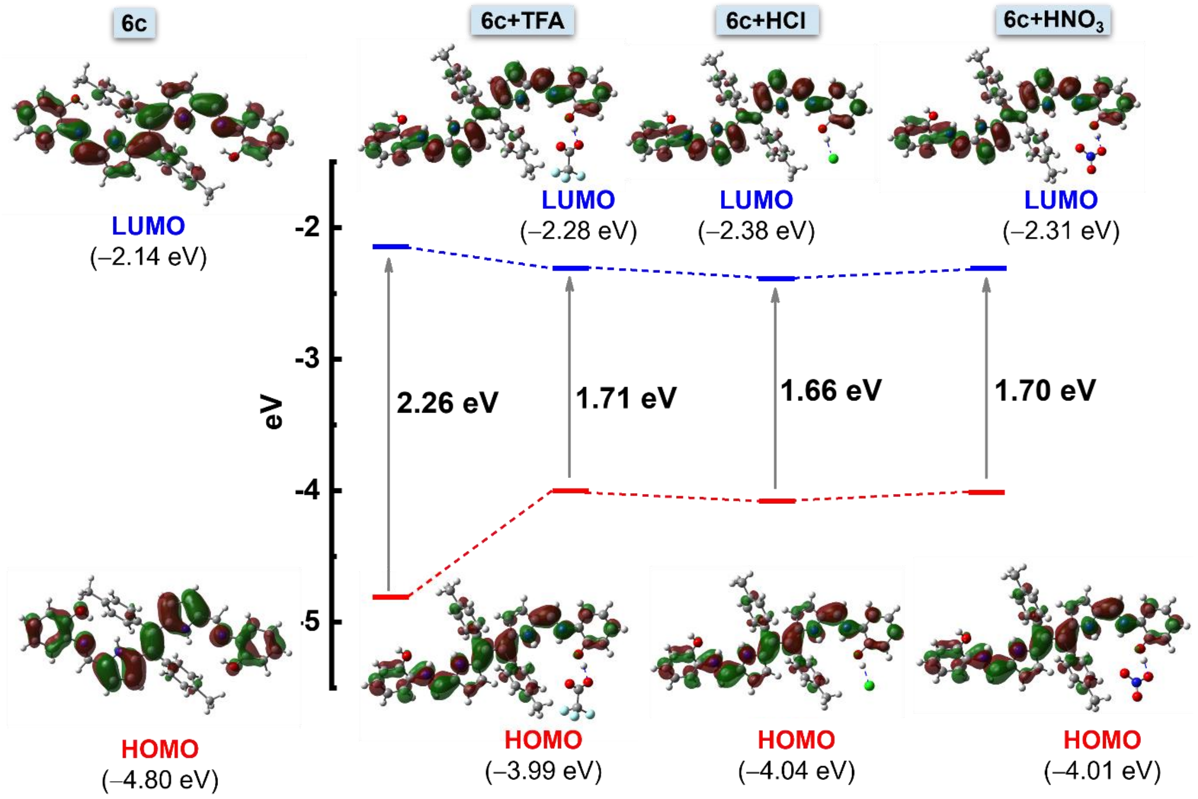

Figure S16 Selected frontier molecular orbital with energy level diagrams of compound **6c** and **6c**+TFA, **6c**+HCl, **6c**+HNO_3.

TD-DFT and Natural Transition Orbit analysis of 6a, 6b and 6c

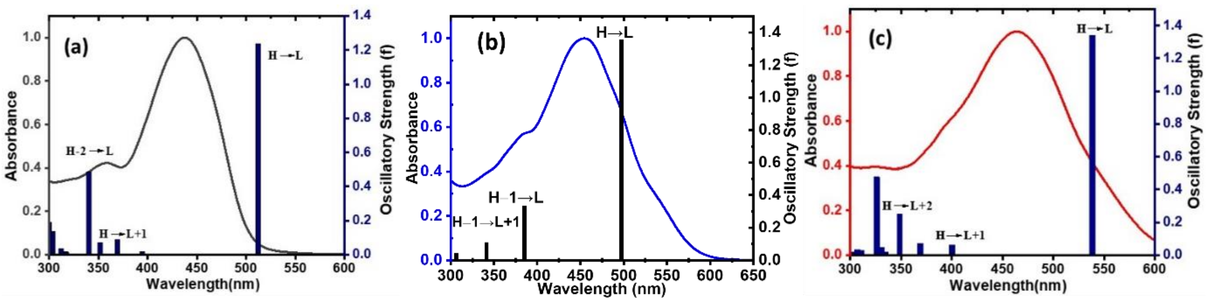

Figure S17-A The calculated excitations (blue vertical lines) are overlaid with the experimental absorption spectra: black for **6a**, sky blue for **6b**, and red for **6c**.

Comp.	λ_{nm}, cal		Hole	Electron
**6c**	538.30	S1 (W= 0.99)	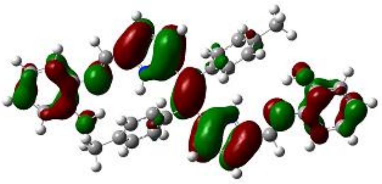	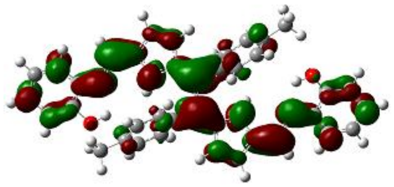
	369.06	S4 (W= 0.89)	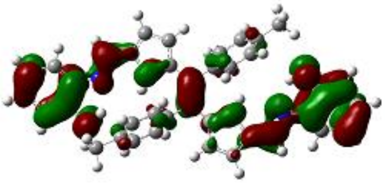	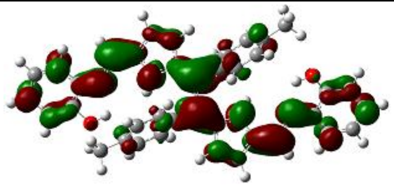
	335. 03	S6 (W=0.90)	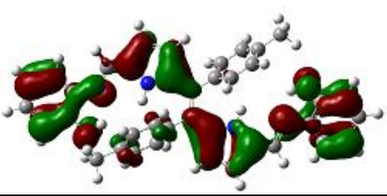	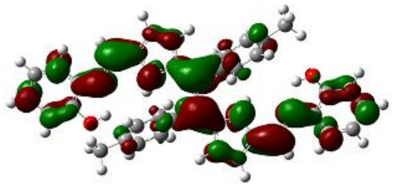
**6b**	497.45	S1 (W=0.98)	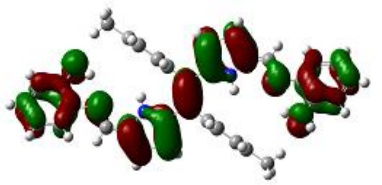	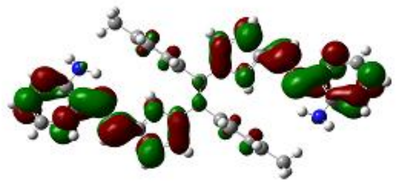
	385.02	S3 (W=0.97)	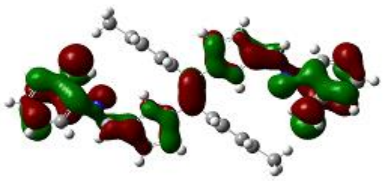	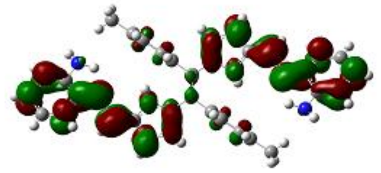
	341.42	S5 (W=0.94)	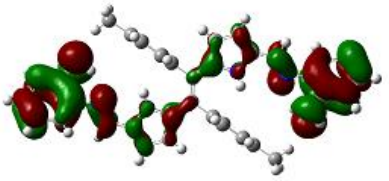	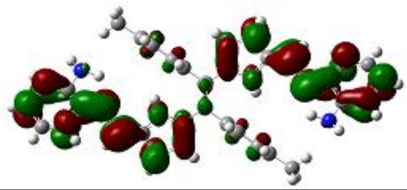
**6a**	512.16	S1 (W=0.99)	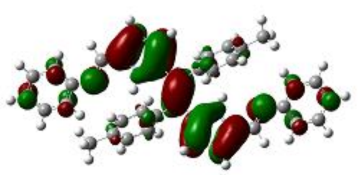	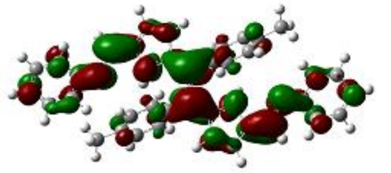
	394.45	S2 (W=0.84)	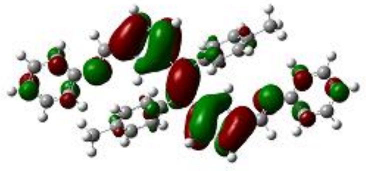	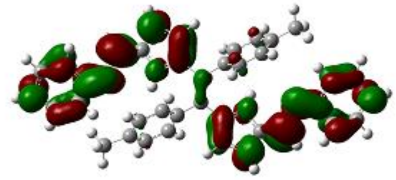

	314.37	S7 (W=0.94)	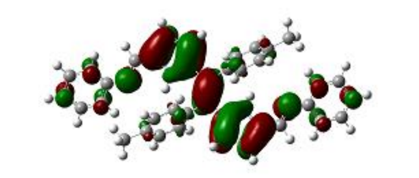	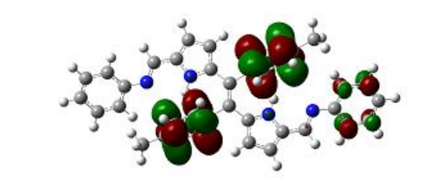

Figure S17-B Natural transition orbitals (NTOs) of compounds **6c**, **6b**, and **6a** are shown in only occupied (holes) and unoccupied (electrons) NTO pairs that contribute more than 50% to the excited state.

^{1}H NMR Spectra

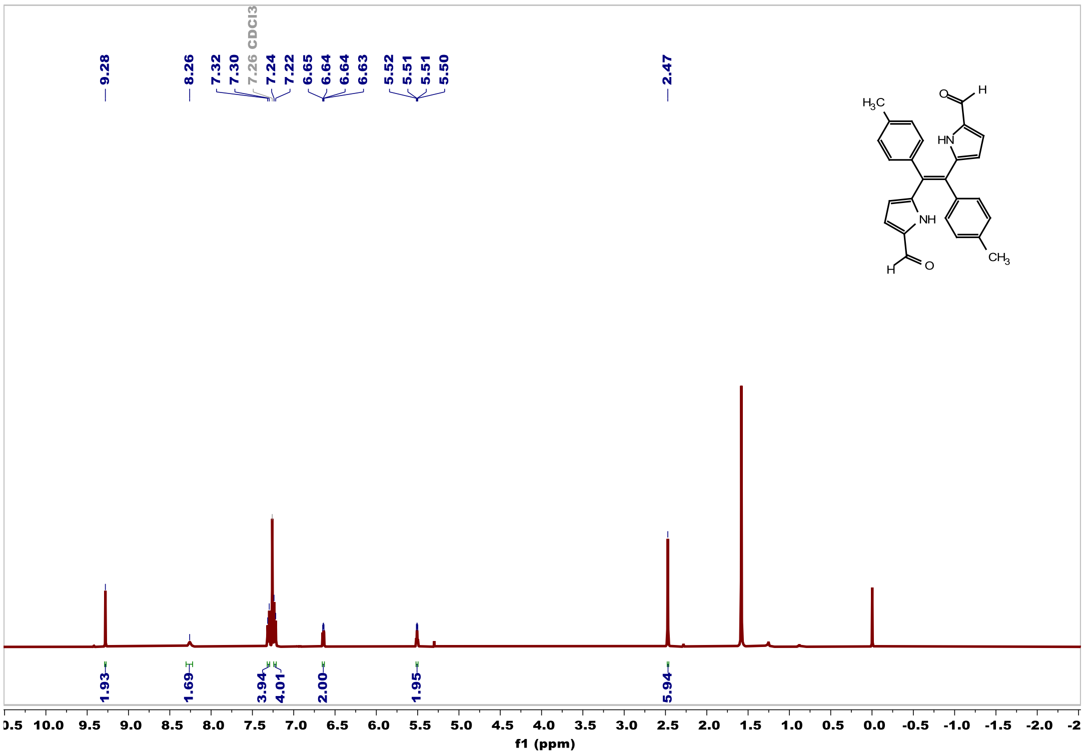

Figure S18 ^{1}H NMR spectrum of compound **5** in $CDCl_3$ at room temperature.

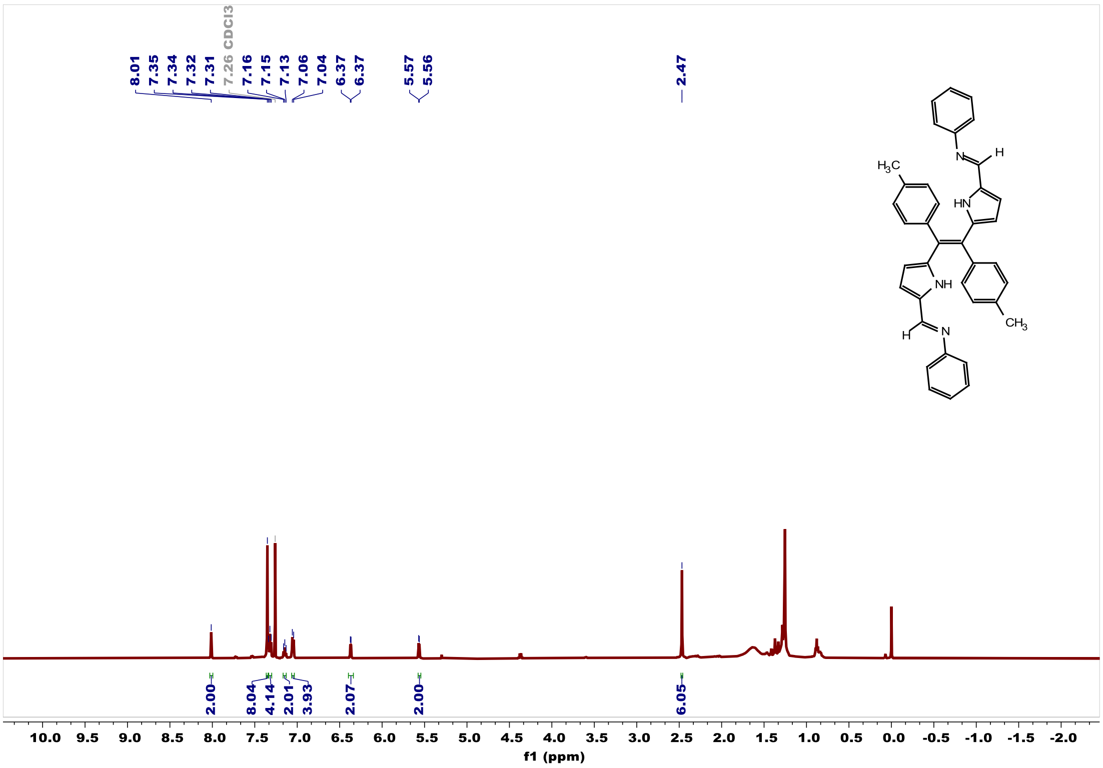

Figure S19 ^{1}H NMR spectrum of compound **6a** in $CDCl_3$ at room temperature.

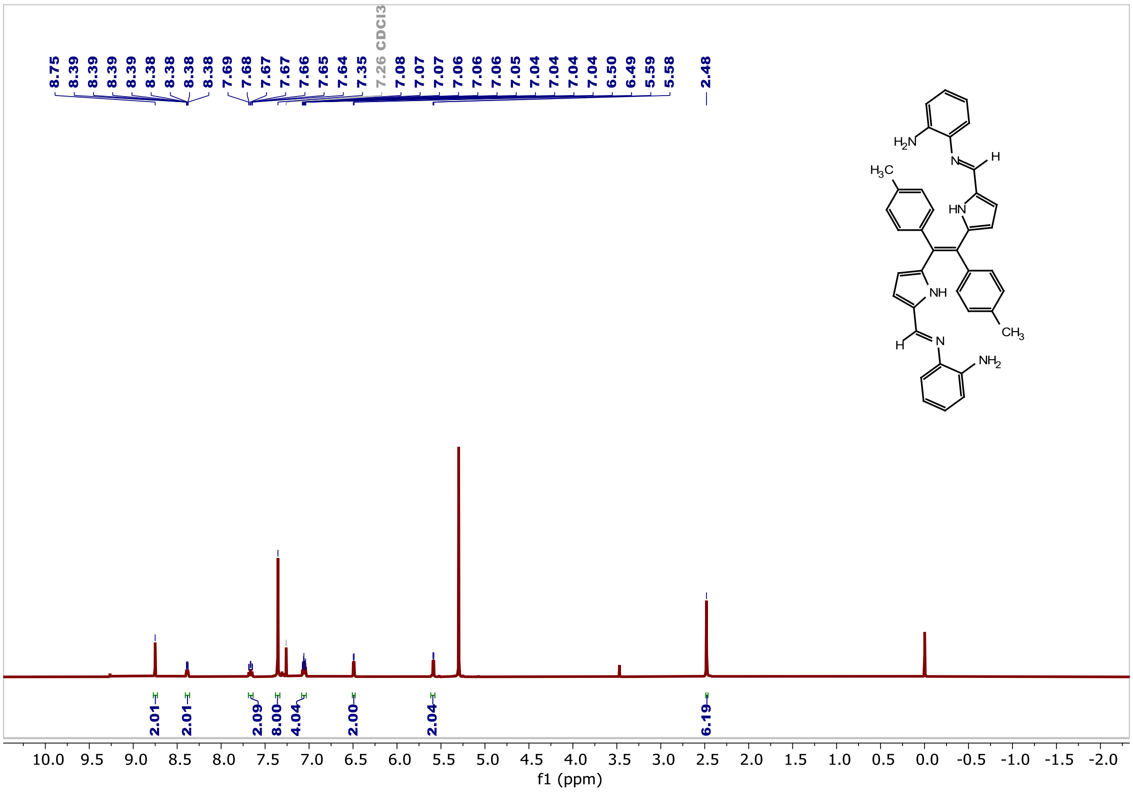

Figure S20 ^{1}H NMR spectrum of compound **6b** in $CDCl_3$ at room temperature.

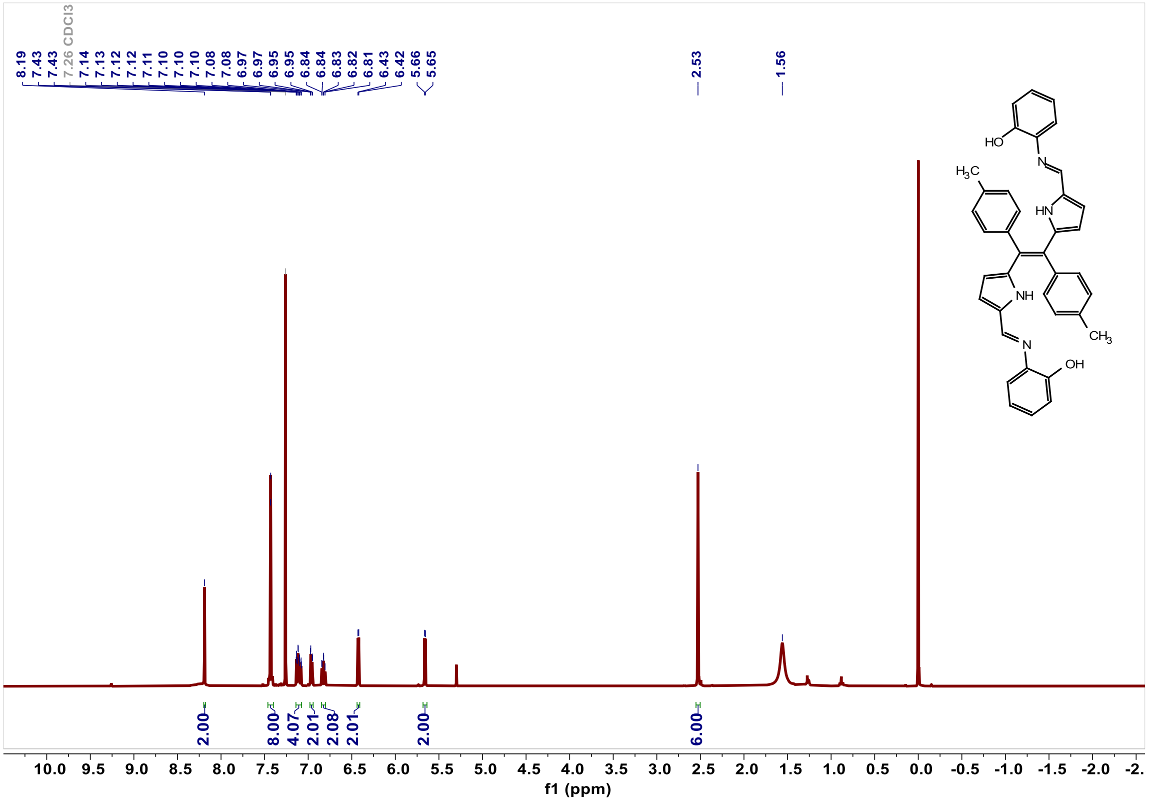

Figure S21 ^{1}H NMR spectrum of compound **6c** in $CDCl_3$ at room temperature.

^{13}C NMR Spectra

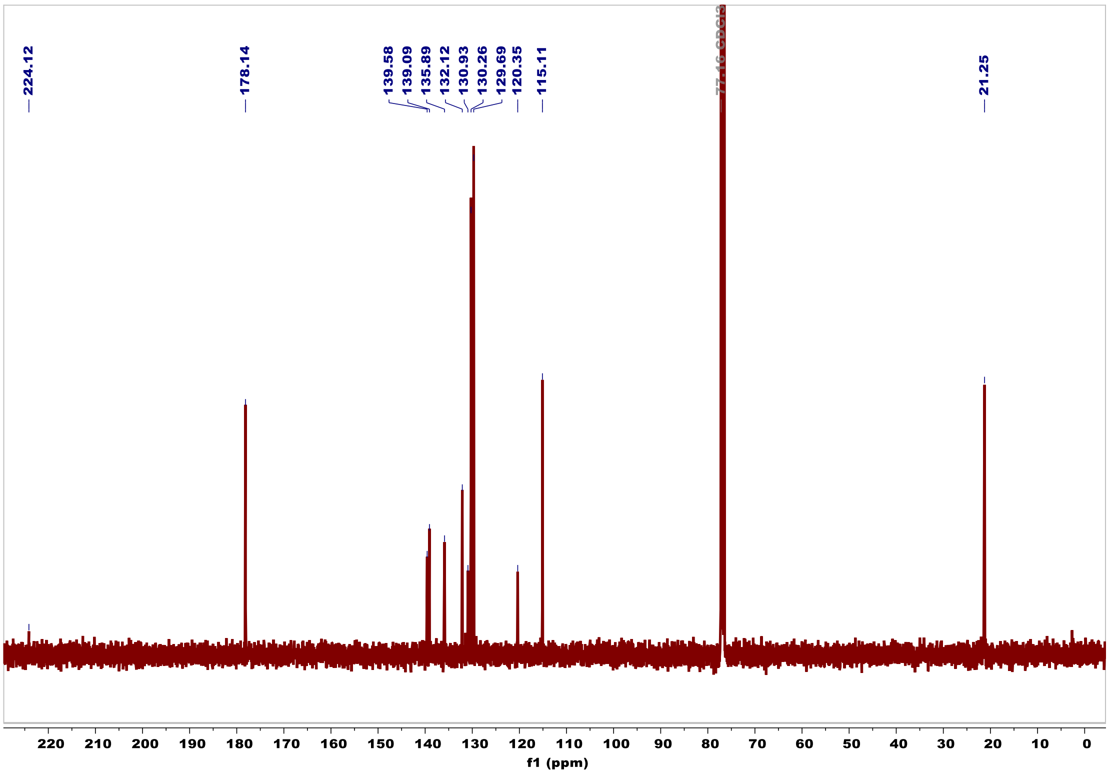

Figure S22 ^{13}C NMR spectrum of compound **5** in $CDCl_3$ at room temperature.

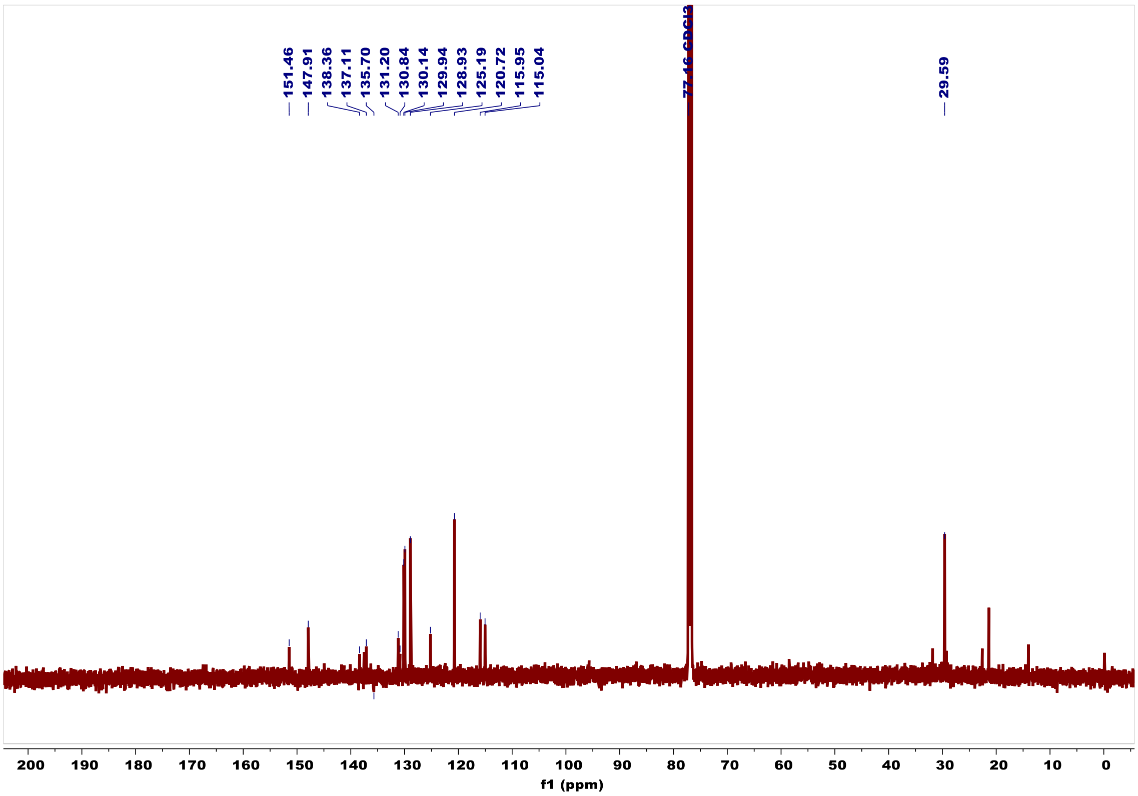

Figure S23 ^{13}C NMR spectrum of compound **6a** in $CDCl_3$ at room temperature.

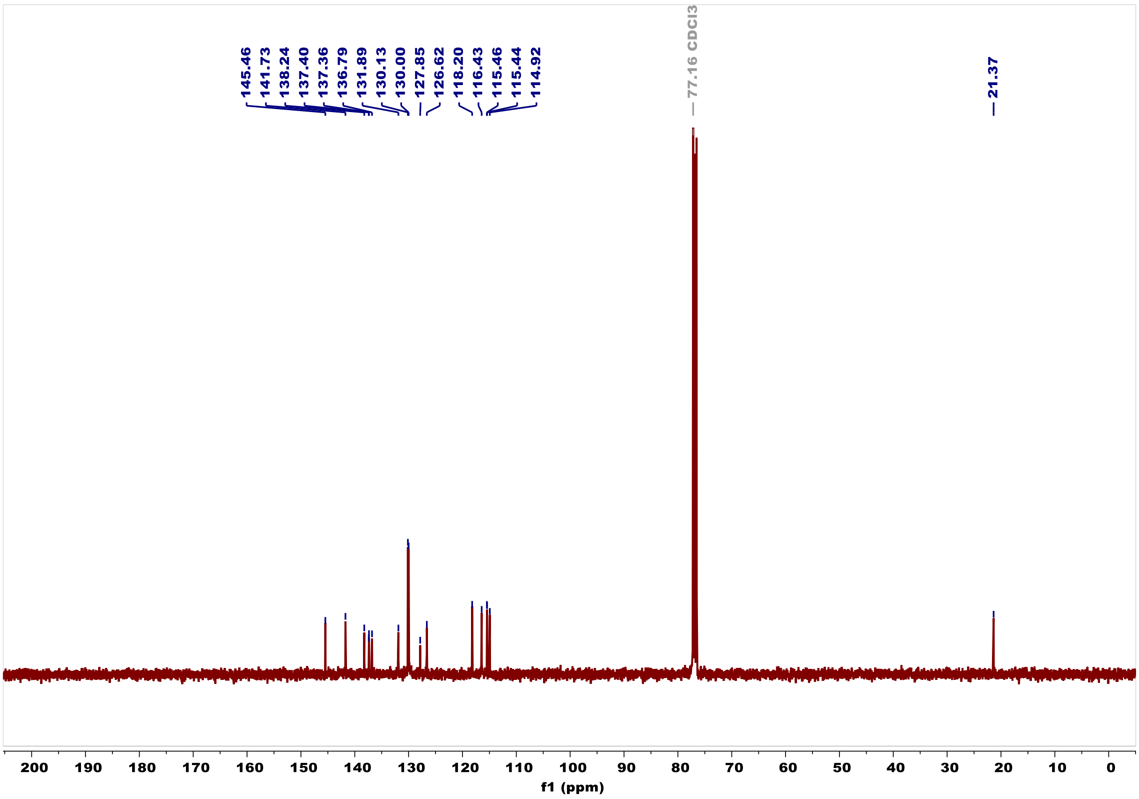

Figure S24 ^{13}C NMR spectrum of compound **6b** in $CDCl_3$ at room temperature.

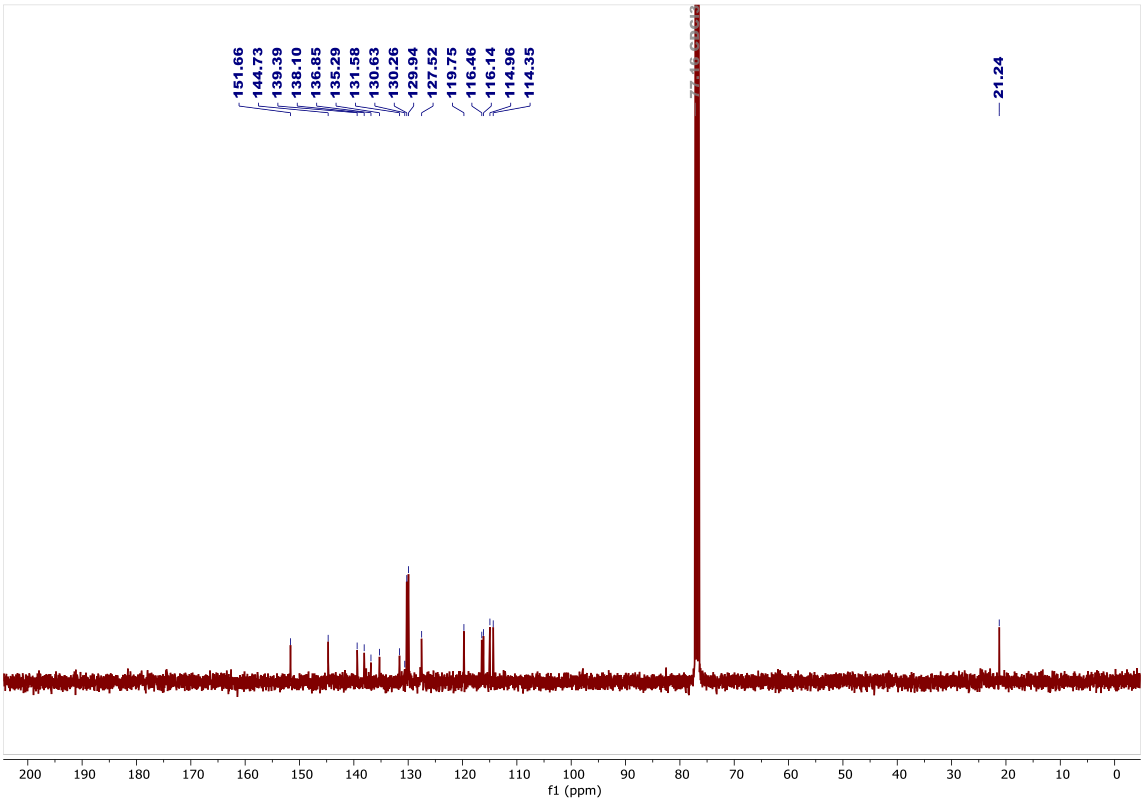

Figure S25 ^{13}C NMR spectrum of compound **6c** in $CDCl_3$ at room temperature.

^{1}H-^{1}H COSY Spectra

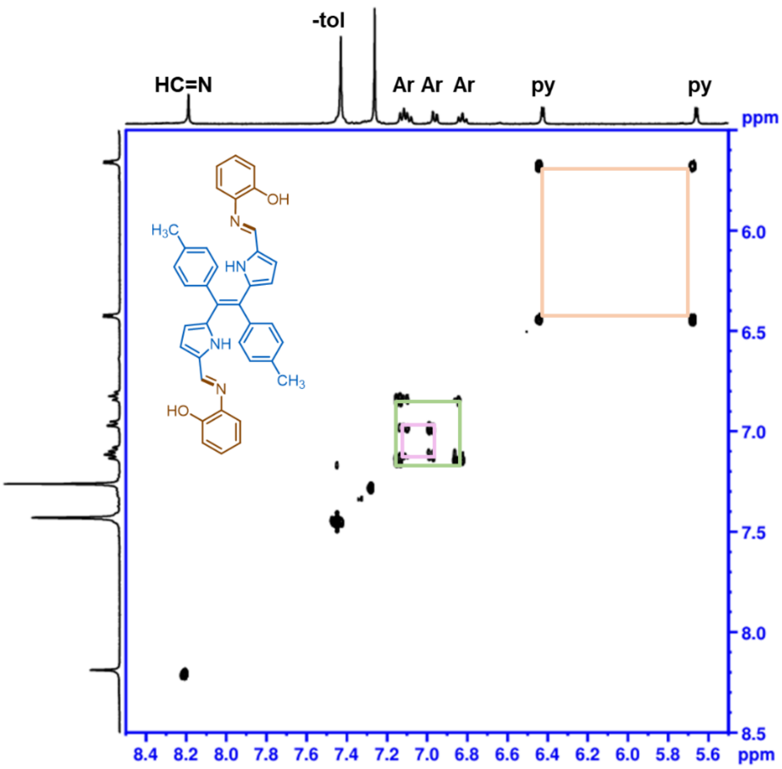

Figure S26 ^{1}H-^{1}H COSY spectrum of the **6c** in $CDCl_3$ at room temperature.

HRMS Spectra

Compound Details

Cpd. 1: C26 H22 N2 O2

Formula	m/z	Observed M/Z	Difference_Da	Difference_PPM	Score
C26 H22 N2 O2	395.1750	395.175041238921	-0.269532039283149	-0.683799678774841	99.56

Compound Spectra (Zoomed)

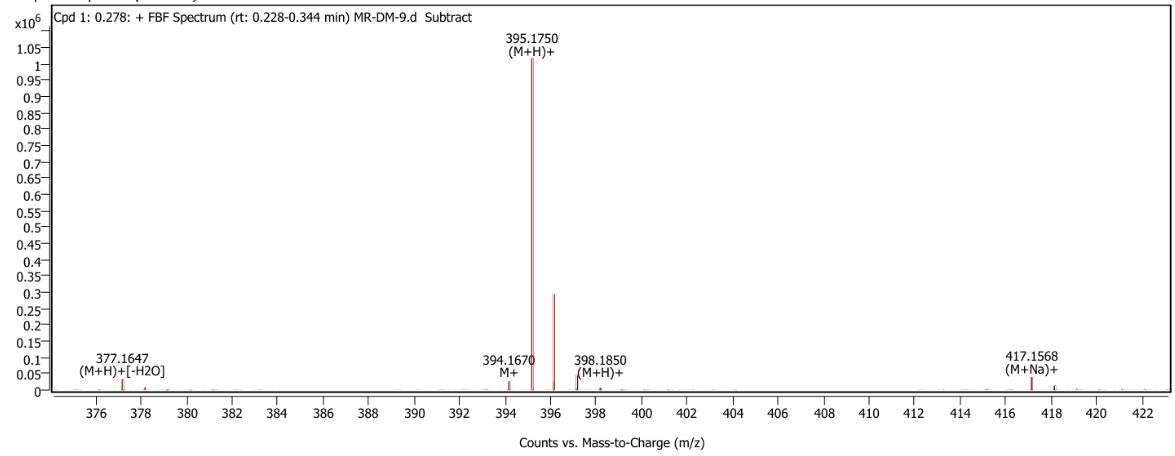

Figure S27 High-resolution mass spectrum of **5**

Compound Details

Cpd. 1: C38 H32 N4

Formula	m/z	Observed M/Z	Difference_Da	Difference_PPM	Score
C38 H32 N4	545.2712	545.27116445582	1.11894831525206	2.05589749456674	96.23

Compound Spectra (Zoomed)

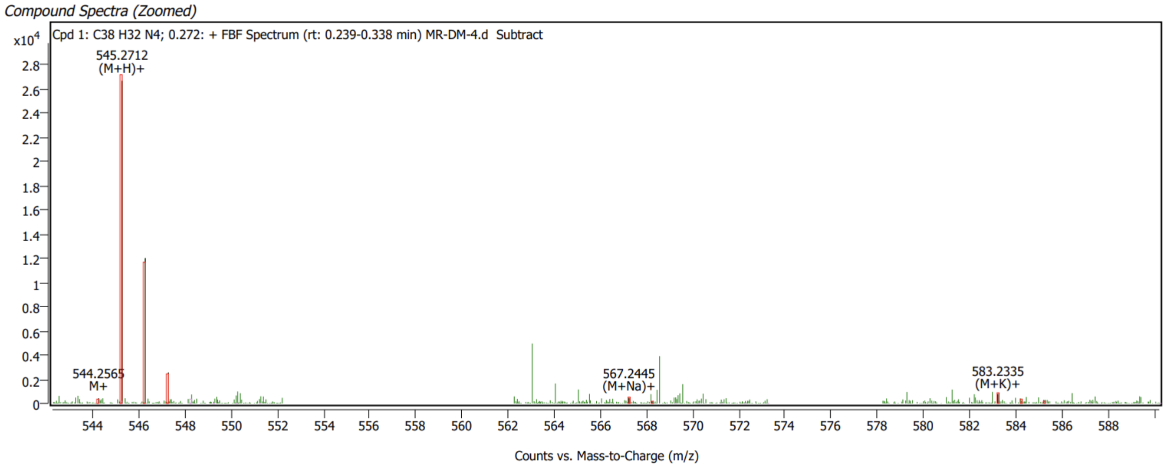

Figure S28 High-resolution mass spectrum of **6a**

Compound Details

Cpd. 1: C38 H34 N6

Formula	m/z	Observed M/Z	Difference_Da	Difference_PPM	Score
C38 H34 N6	575.2929	575.292915292356	1.00560085309098	1.7510499789222	96.83

Department of Chemistry I.I.T. (B)

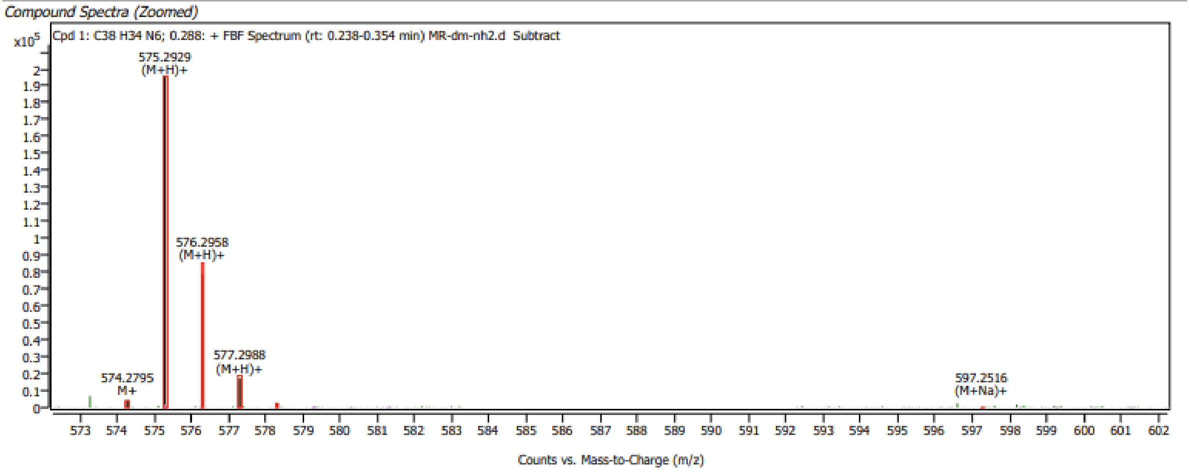

Figure S29 High resolution mass spectrum of **6b**

Compound Details

Cpd. 1: C38 H32 N4 O2

Formula	m/z	Observed M/Z	Difference_Da	Difference_PPM	Score
C38 H32 N4 O2	577.2593	577.25925386928	-0.432561225352401	-0.750645256400943	99.38

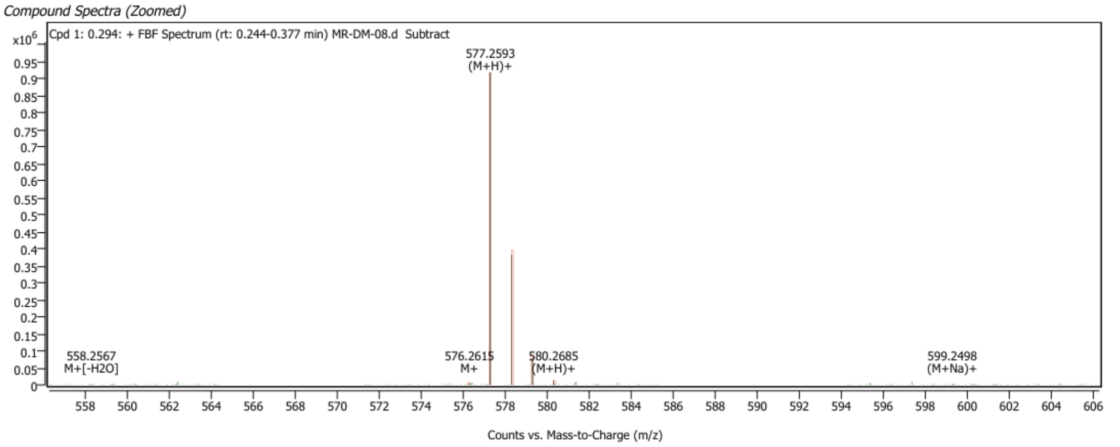

Figure S30 High resolution mass spectrum of **6c.**

Compound Details

Cpd. 1: C42 H34 F6 N4 O6

Formula	m/z	Observed M/Z	Difference_Da	Difference_PPM	Score
C42 H34 F6 N4 O6	804.2293	804.229256269044	-7.3788299895341	-9.17493037964059	49.85

Compound Spectra (Zoomed)

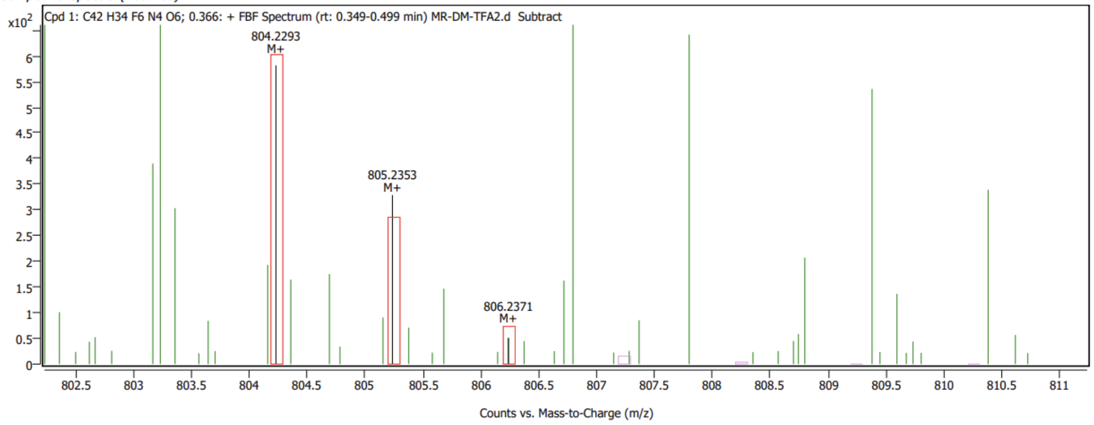

Figure S31 High resolution mass spectrum of **6c+2H$^+$.**

DFT- Optimized XYZ Coordinates

Table S9 S_0 optimized geometry of the compound **6a** at B3LYP/6-31g (d,p)

Atom	X	Y	Z	Atom	X	Y	Z
N	-2.38035	1.173529	-0.07655	C	7.217522	-1.804	-1.42053
N	-5.06077	0.808061	-0.68551	C	8.372884	0.696704	-0.94532
C	-1.13293	1.599686	0.297924	C	9.135593	-0.32416	-1.52275
C	0.024701	0.749362	0.531421	C	-5.32111	-2.98801	1.020638
C	1.31868	1.486828	0.621456	C	8.548434	-1.57011	-1.76415
C	-1.20148	3.004919	0.375132	H	-2.685	0.220919	-0.21179
C	1.818508	2.214609	-0.46869	H	-0.37285	3.643146	0.643196
C	-2.51027	3.39774	0.052884	H	1.228865	2.277159	-1.37808
C	3.850136	2.747431	0.761433	H	-2.9057	4.404219	0.028423
C	-4.61713	2.012975	-0.54102	H	-5.2457	2.905562	-0.64951
C	-3.23601	2.233884	-0.22073	H	3.929363	1.96056	2.764995
C	3.337946	2.036854	1.856283	H	1.719247	0.845037	2.633729
C	2.09217	1.417889	1.790333	H	3.453287	3.366891	-1.26186
C	3.070133	2.827699	-0.39943	H	-7.23776	2.304693	0.064019
C	-6.41804	0.556379	-0.91227	H	-8.30095	-1.9176	-2.32687
C	-6.74223	-0.64976	-1.56189	H	-10.139	-0.40503	-1.59295
C	-7.46608	1.397515	-0.48715	H	5.098221	4.442394	1.252636
C	-8.07034	-0.98739	-1.81523	H	5.880254	2.875295	1.49127
C	-9.1032	-0.13791	-1.40602	H	5.651144	3.517657	-0.15009
C	5.194415	3.428863	0.84112	H	-9.59012	1.704816	-0.39552

C	-8.79271	1.05019	-0.73645	H	2.708594	-0.23648	-0.12297
N	2.399904	-1.14969	0.176613	H	0.370391	-3.43695	1.317768
N	5.104543	-0.9275	-0.45806	H	-1.7683	-0.43747	2.702423
C	1.145387	-1.4985	0.605349	H	2.916324	-4.29866	0.913729
C	-0.01515	-0.62401	0.6496	H	5.266597	-2.97007	0.016217
C	-1.31703	-1.33622	0.802404	H	-3.44046	-3.37814	-0.91768
C	1.207395	-2.86201	0.950385	H	-1.16728	-2.39651	-1.06455
C	-2.13041	-1.09721	1.919883	H	-4.02801	-1.44652	2.868896
C	2.522225	-3.30674	0.738357	H	6.758432	-2.76351	-1.63932
C	-3.90774	-2.4646	0.975866	H	8.817147	1.671479	-0.76343
C	4.641627	-2.0738	-0.08474	H	10.17165	-0.14677	-1.79547
C	3.255226	-2.21759	0.257295	H	-5.68253	-3.07029	2.050871
C	-3.07493	-2.73923	-0.11769	H	-5.39679	-3.97167	0.544437
C	-1.79489	-2.19093	-0.20278	H	-5.99504	-2.3076	0.483057
C	-3.40422	-1.65526	2.0037	H	9.12756	-2.36135	-2.23238
C	6.448998	-0.78571	-0.823	H	6.429445	1.254838	-0.16995
C	7.039403	0.473312	-0.61025	H	-5.92732	-1.29908	-1.86685

Table S10 S_0 optimized geometry of the compound **6b** at B3LYP/6-31g (d,p)

Atom	X	Y	Z	Atom	X	Y	Z
N	1.463213	-0.22493	-1.0695	N	-1.60208	-0.11763	1.261311
H	1.904134	-0.20469	-0.16135	H	-1.92062	-0.28179	0.317692
N	3.948294	-1.45325	-0.86871	N	-3.96036	-1.49781	0.96081
N	5.819558	-0.25624	0.742696	N	-5.40499	-0.83582	-1.26836
H	4.838441	-0.00295	0.704826	H	-4.73434	-0.21704	-0.83119
H	6.235665	-0.08706	1.648643	H	-6.12408	-0.3471	-1.78345
C	-1.51429	1.988001	-0.88738	C	1.463019	1.912771	0.950365
N	-0.29189	1.330178	-0.59515	N	0.168624	1.385031	0.719497
C	3.315659	-1.51104	-1.99556	C	-3.49853	-1.34216	2.159024
H	3.723219	-2.01827	-2.87855	H	-4.00233	-1.7304	3.052514
C	0.392943	0.502893	-1.48635	C	-0.59836	0.704088	1.665319
C	2.057376	-0.85001	-2.15432	C	-2.29808	-0.58924	2.36033
C	-1.79239	3.216628	-0.27564	C	2.29036	1.38273	1.952056
H	-1.05585	3.659103	0.386739	H	1.920185	0.602843	2.605888
C	-2.4791	1.388392	-1.70747	C	1.966014	2.899208	0.092385
H	-2.291	0.416934	-2.15	H	1.333547	3.292527	-0.69609
C	1.293067	-0.53705	-3.27304	C	-1.67121	-0.07315	3.490309
H	1.487129	-0.87977	-4.28034	H	-1.97016	-0.25804	4.513183
C	0.247278	0.322476	-2.86478	C	-0.60601	0.753196	3.061871
H	-0.51489	0.768935	-3.48289	H	0.069937	1.326602	3.676499
C	6.098738	-1.50725	0.197044	C	-5.86462	-1.8601	-0.44673
C	5.169917	-2.11237	-0.69374	C	-5.12257	-2.23248	0.709347
C	-3.98422	3.272952	-1.34262	C	4.131599	2.803877	1.2106
C	-3.01531	3.841073	-0.502	C	3.600123	1.842487	2.079719

H	-3.22292	4.791243	-0.01704	H	4.230667	1.418564	2.857353
C	-3.6919	2.039825	-1.93658	C	3.284282	3.327909	0.225294
H	-4.42903	1.565583	-2.57907	H	3.668264	4.077501	-0.4612
C	7.316086	-2.15601	0.453003	C	-7.04374	-2.5587	-0.74912
H	8.026689	-1.68728	1.129624	H	-7.61415	-2.26674	-1.62772
C	-5.29271	3.976253	-1.60655	C	5.583616	3.201408	1.296423
H	-5.18408	4.729861	-2.39778	H	6.210871	2.410816	0.863515
H	-5.65207	4.493372	-0.71042	H	5.776013	4.128007	0.746643
H	-6.06345	3.268571	-1.92922	H	5.897975	3.34459	2.336659
C	5.494109	-3.34206	-1.28911	C	-5.5699	-3.30906	1.492353
H	4.756846	-3.81631	-1.93096	H	-4.97018	-3.61603	2.344775
C	7.619325	-3.37574	-0.1514	C	-7.47797	-3.61415	0.052014
H	8.56549	-3.86144	0.070093	H	-8.39039	-4.14269	-0.20941
C	6.708994	-3.97494	-1.02769	C	-6.73851	-3.99975	1.17484
H	6.933993	-4.93292	-1.48657	H	-7.06071	-4.83661	1.786937

Table S11 S_0 optimized geometry of the compound **6c** at B3LYP/6-31g (d,p)

Atom	X	Y	Z	Atom	X	Y	Z
O	6.474062	1.112039	0.407185	C	-6.80497	-0.6138	-1.23961
N	2.396194	-1.19865	0.07498	C	-7.56856	1.569685	-0.50252
N	5.190475	-1.10844	-0.36451	C	-8.1223	-0.9867	-1.51157
C	1.114651	-1.52359	0.44216	C	-9.15678	-0.07957	-1.28201
C	-0.0106	-0.61344	0.559969	C	4.942179	3.922464	0.32519
C	-1.31448	-1.29839	0.814121	C	-8.88299	1.19664	-0.7736
C	1.098701	-2.91866	0.640394	H	5.545111	0.808591	0.408893
C	-1.92376	-2.09216	-0.17036	H	2.747229	-0.27555	-0.12844
C	2.386111	-3.41139	0.386879	H	0.227979	-3.48406	0.935352
C	-3.88773	-2.40336	1.238338	H	-1.40553	-2.26256	-1.1092
C	4.590623	-2.24628	-0.23013	H	2.723014	-4.43672	0.45661
C	3.188632	-2.31928	0.043978	H	5.122182	-3.20283	-0.29001
C	-3.25963	-1.63619	2.228297	H	-3.77836	-1.45354	3.165576
C	-1.99395	-1.09031	2.021873	H	-1.53556	-0.46673	2.783271
C	-3.19671	-2.62665	0.036848	H	-3.66956	-3.21383	-0.74576
C	6.556609	-1.04049	-0.65455	H	6.823234	-2.91201	-1.68531
C	7.190019	0.15772	-0.24369	H	9.010482	1.282192	-0.14706
C	7.314937	-2.01205	-1.3275	H	10.34612	-0.4674	-1.32552
C	8.55053	0.357418	-0.48025	H	-5.19534	-4.06024	1.686764
C	9.28871	-0.62842	-1.13753	H	-5.77084	-2.49711	2.289471
C	-5.26058	-2.98899	1.455397	H	-5.86932	-2.87711	0.553472
C	8.673569	-1.81061	-1.56636	H	9.24752	-2.56436	-2.09644
O	-5.79945	-1.50088	-1.46089	H	-4.9847	-1.06205	-1.14465
N	-2.40823	1.204199	0.029514	H	-2.7109	0.255452	-0.12734
N	-5.14781	0.905295	-0.50675	H	-0.35786	3.642803	0.73519
C	-1.13713	1.615924	0.337138	H	1.970428	0.911327	2.358668

C	0.037775	0.758519	0.44278	H	-2.9277	4.421529	0.343046
C	1.322199	1.518959	0.398373	H	-5.28134	2.989617	-0.2657
C	-1.20222	3.014762	0.494119	H	3.0441	3.669034	-1.61648
C	2.208149	1.513937	1.48728	H	0.968263	2.320929	-1.56647
C	-2.53032	3.417024	0.28893	H	4.042731	2.272478	2.317225
C	3.695323	3.073586	0.346892	H	-7.36159	2.550857	-0.08612
C	-4.66672	2.083101	-0.27086	H	-8.31601	-1.98218	-1.89792
C	-3.27308	2.266669	-8.5E-05	H	-10.1814	-0.37251	-1.49133
C	2.815938	3.061653	-0.74461	H	5.166283	4.313941	1.323318
C	1.648662	2.30082	-0.72088	H	4.82776	4.766325	-0.36286
C	3.377958	2.274964	1.457078	H	5.805904	3.32953	0.005428
C	-6.50719	0.679306	-0.73953	H	-9.6932	1.893042	-0.58091

Table S12 S_0 optimized geometry of the compound **6c**+TFA at B3LYP/6-31g (d,p)

Atom	X	Y	Z	Atom	X	Y	Z
O	5.938468	0.361605	-0.801	H	-4.42214	0.389117	-0.09719
H	6.358985	-0.60464	-0.71531	N	-6.71159	-1.25777	-0.05563
N	1.801732	1.957559	-0.1235	H	-6.45994	-0.43421	0.475282
H	2.281628	1.176715	0.300728	C	-2.52242	2.034113	-0.06895
N	4.624032	2.487009	-0.21737	C	-5.71385	-2.06361	-0.52118
H	4.335927	1.521716	-0.37745	H	-5.99152	-3.06195	-0.83201
C	0.402554	-0.46207	0.203458	C	-4.38926	-1.69307	-0.58394
C	3.717506	3.464389	-0.3239	C	-2.50082	-0.41333	-0.41917
H	4.080339	4.482854	-0.40129	C	-8.08161	-1.52598	-0.10632
C	2.352687	3.204321	-0.36675	C	-1.74682	0.771708	-0.22887
C	0.441821	1.958355	-0.35142	C	-8.93542	-0.71443	0.673574
C	6.011586	2.570345	-0.05284	C	-2.38168	2.805394	1.09597
C	-0.33711	0.779956	-0.15877	H	-1.69938	2.467133	1.86965
C	6.693881	1.357461	-0.33942	C	-2.12791	-1.73548	-0.83206
C	0.069217	-1.13686	1.388256	H	-1.11629	-2.05119	-1.03031
H	-0.73948	-0.75198	2.0021	C	-8.64256	-2.54197	-0.89016
C	0.124059	3.285679	-0.78627	H	-8.00412	-3.14722	-1.52385
H	-0.85659	3.623203	-1.07852	C	-3.3968	2.490367	-1.06781
C	6.696191	3.703242	0.389478	H	-3.50598	1.912509	-1.98122
H	6.149647	4.611744	0.624933	C	-10.3089	-0.93345	0.673787
C	1.449202	-0.98023	-0.58254	H	-10.9462	-0.29836	1.284421
H	1.717429	-0.47884	-1.50841	C	-3.08329	3.997318	1.249873
C	8.078817	1.304474	-0.13635	H	-2.95629	4.583075	2.156814
H	8.598217	0.372587	-0.33462	C	-3.2634	-2.49974	-0.93941
C	0.75667	-2.28488	1.771908	H	-3.32668	-3.53976	-1.23031
H	0.487678	-2.78849	2.696982	C	-10.0215	-2.76313	-0.87895
C	1.278844	4.040508	-0.77447	H	-10.4381	-3.55581	-1.49177
H	1.384513	5.07583	-1.06971	C	-3.95135	4.461126	0.249261
C	8.08052	3.639753	0.557092	C	-10.8567	-1.96318	-0.09922

H	8.622296	4.514066	0.903038	H	-11.929	-2.12852	-0.09148
C	1.805368	-2.79813	0.996618	C	-4.09576	3.688726	-0.91012
C	8.760336	2.441339	0.303946	H	-4.75446	4.035607	-1.70219
H	9.835129	2.391267	0.452344	C	-4.72342	5.744811	0.431055
C	2.140016	-2.12615	-0.18952	H	-5.62366	5.57865	1.037544
H	2.953242	-2.50874	-0.79752	H	-4.11989	6.499589	0.946597
C	2.588653	-4.00385	1.440476	H	-5.04399	6.15443	-0.53209
H	3.592072	-3.69053	1.746167	O	5.255515	-2.12093	1.045331
H	2.094452	-4.51265	2.274796	O	6.722778	-1.97546	-0.68412
H	2.723084	-4.71418	0.618826	F	5.648735	-4.81751	0.992735
O	-8.32152	0.271072	1.404335	F	4.721548	-4.3208	-0.91854
H	-8.98419	0.777256	1.892344	F	6.875436	-4.6357	-0.7901
N	-3.87781	-0.44264	-0.26447	C	5.95448	-2.57418	0.134558
				C	5.824085	-4.10195	-0.13988

Table S13 S_0 optimized geometry of the compound **6c**+HCl at B3LYP/6-31g (d,p)

Atom	X	Y	Z	Atom	X	Y	Z
O	6.194574	-1.0212	-0.0795	O	-7.60494	0.620853	1.574323
H	6.517685	-1.98254	0.064324	H	-8.18979	1.195487	2.085505
N	2.53718	1.264713	-0.20432	N	-3.33657	-0.54923	-0.27587
H	2.909662	0.415438	0.193006	H	-3.78087	0.334527	-0.08154
N	5.370738	1.404542	-0.18655	N	-6.2299	-1.05295	0.034657
H	4.918412	0.49381	-0.2323	H	-5.86987	-0.26419	0.555862
C	0.976615	-1.00116	-0.09818	C	-1.73186	1.768826	-0.10347
C	4.627162	2.503799	-0.35933	C	-5.34537	-1.95992	-0.46942
H	5.135739	3.455071	-0.45699	H	-5.74317	-2.91894	-0.77359
C	3.239001	2.435316	-0.42613	C	-3.9899	-1.73532	-0.57906
C	1.190586	1.42806	-0.43857	C	-1.97121	-0.66548	-0.48256
C	6.74995	1.248453	-0.00614	C	-7.62254	-1.16634	0.032966
C	0.305353	0.315779	-0.30561	C	-1.0944	0.436608	-0.31045
C	7.167791	-0.10701	0.059483	C	-8.35082	-0.27812	0.855207
C	0.773635	-1.72239	1.086896	C	-1.49593	2.491363	1.077145
H	0.063262	-1.34787	1.818011	H	-0.84545	2.064026	1.833916
C	1.03178	2.795116	-0.83185	C	-1.75654	-2.01509	-0.91329
H	0.096335	3.253241	-1.10987	H	-0.79383	-2.43565	-1.15428
C	7.667399	2.293322	0.119885	C	-8.32137	-2.09999	-0.74223
H	7.329573	3.324603	0.081466	H	-7.77773	-2.7611	-1.40783
C	1.897499	-1.50681	-1.03474	C	-2.56491	2.337975	-1.07862
H	2.066918	-0.96008	-1.95796	H	-2.74644	1.796901	-2.00298
C	8.523288	-0.39065	0.257299	C	-9.73932	-0.34316	0.905249
H	8.82884	-1.43038	0.314592	H	-10.2781	0.348608	1.548317
C	1.483878	-2.89727	1.334809	C	-2.06647	3.745987	1.268678
H	1.3271	-3.43277	2.267538	H	-1.86826	4.292147	2.187499
C	2.272929	3.40282	-0.81612	C	-2.97151	-2.65433	-0.97899

H	2.500653	4.423758	-1.09199	H	-3.15607	-3.67963	-1.2703
C	9.017224	1.994803	0.307649	C	-9.71497	-2.16638	-0.68136
H	9.737472	2.800473	0.406509	H	-10.2399	-2.89696	-1.28814
C	2.421874	-3.38947	0.416586	C	-2.8926	4.323003	0.291473
C	9.435971	0.659692	0.377275	C	-10.4265	-1.29199	0.140323
H	10.48697	0.432625	0.529527	H	-11.5095	-1.33713	0.186744
C	2.607698	-2.67727	-0.7791	C	-3.13188	3.599214	-0.88314
H	3.331988	-3.03809	-1.50393	H	-3.7604	4.032441	-1.6569
C	3.235202	-4.62606	0.698899	C	-3.51942	5.677353	0.514783
H	4.30964	-4.39627	0.683392	H	-4.37465	5.605424	1.199481
H	2.980131	-5.05196	1.674429	H	-2.8036	6.374804	0.963597
H	3.059301	-5.39202	-0.06682	H	-3.88007	6.107218	-0.42485
				Cl	7.023929	-3.78069	0.340873

Table S14 S_0 optimized geometry of the compound **6c**+HNO_3 at B3LYP/6-31g (d,p)

Atom	X	Y	Z	Atom	X	Y	Z
O	6.440573	-0.55739	-0.9308	N	-3.56237	-0.47985	-0.31012
H	6.715665	-1.51511	-0.6414	H	-4.0075	0.392803	-0.07282
N	2.330819	1.334855	-0.27998	N	-6.45449	-0.99698	-0.01928
H	2.744697	0.490211	0.088396	H	-6.09361	-0.24168	0.548882
N	5.208254	1.63846	-0.34888	C	-1.95283	1.830235	-0.05676
H	4.860657	0.696384	-0.52662	C	-5.57113	-1.87826	-0.57065
C	0.717728	-0.95617	-0.06719	H	-5.97082	-2.8202	-0.92233
C	4.372462	2.680111	-0.43327	C	-4.21682	-1.6495	-0.67115
H	4.809644	3.670829	-0.47822	C	-2.1976	-0.58488	-0.52557
C	2.99227	2.53394	-0.48213	C	-7.84701	-1.10919	-0.02753
C	0.972023	1.476485	-0.46895	C	-1.31783	0.506045	-0.31123
C	6.590458	1.634272	-0.12423	C	-8.57502	-0.27027	0.845242
C	0.086242	0.37091	-0.30805	C	-1.69075	2.522744	1.13654
C	7.217938	0.390933	-0.39278	H	-1.02097	2.077416	1.865635
C	0.39049	-1.68394	1.087148	C	-1.98413	-1.91329	-1.02247
H	-0.34661	-1.27831	1.77363	H	-1.02074	-2.3209	-1.28324
C	0.77256	2.846697	-0.83562	C	-8.54653	-1.9956	-0.85573
H	-0.17878	3.288105	-1.08307	H	-8.00314	-2.61704	-1.55879
C	7.314978	2.717307	0.378457	C	-2.81012	2.423469	-0.99658
H	6.811838	3.65352	0.600737	H	-3.01112	1.906942	-1.93088
C	1.669567	-1.50204	-0.94805	C	-9.96355	-0.33725	0.891535
H	1.931531	-0.95764	-1.85095	H	-10.5022	0.315669	1.574246
C	8.578689	0.246894	-0.09828	C	-2.25903	3.770465	1.374224
H	9.050788	-0.71419	-0.27387	H	-2.03998	4.292995	2.302018
C	0.996599	-2.90984	1.351744	C	-3.19823	-2.54794	-1.118
H	0.736507	-3.45334	2.256397	H	-3.38343	-3.55757	-1.45938
C	1.994346	3.485809	-0.82643	C	-9.94023	-2.06492	-0.7985
H	2.192628	4.519346	-1.07684	H	-10.4654	-2.75874	-1.44677

C	8.679318	2.566902	0.630129	C	-3.10934	4.370835	0.43255
H	9.249371	3.402506	1.023181	C	-10.6513	-1.23965	0.072761
C	1.955572	-3.45033	0.484209	H	-11.7344	-1.28697	0.116697
C	9.300072	1.332001	0.404942	C	-3.37483	3.677346	-0.75487
H	10.35711	1.2123	0.623534	H	-4.02165	4.129392	-1.50241
C	2.277793	-2.72458	-0.67272	C	-3.73483	5.716535	0.705989
H	3.025556	-3.11885	-1.3541	H	-4.59495	5.61924	1.381518
C	2.65554	-4.74621	0.794851	H	-3.02068	6.394079	1.186564
H	3.702243	-4.54988	1.057022	H	-4.08926	6.184026	-0.218
H	2.171682	-5.26793	1.627309	O	5.156972	-2.58453	0.809322
H	2.656782	-5.41153	-0.077	O	7.071737	-2.88841	-0.21654
O	-7.82836	0.584553	1.615624	N	6.104819	-3.34359	0.51283
H	-8.41227	1.121676	2.166943	O	6.152902	-4.52729	0.889937

Table S15. Major transitions were calculated using TD-DFT studies of **6a**.

Wavelength (nm)	Osc. Strength	Major contribs
512.16206631	1.2385	HOMO->LUMO (99%)
394.452128443	0.0159	H-1->LUMO (17%), HOMO->L+1 (82%)
369.06647917	0.0877	H-1->LUMO (78%), HOMO->L+1 (16%)
351.628454374	0.0714	H-2->LUMO (20%), HOMO->L+2 (76%)
340.092695337	0.4848	H-2->LUMO (71%), HOMO->L+2 (19%)
317.184356244	0.0153	H-3->LUMO (79%)
314.369514978	0.0001	HOMO->L+3 (95%)
312.153359884	0.0348	H-4->LUMO (78%)
310.60498788	0.0018	HOMO->L+4 (96%)
303.964777298	0.1384	H-5->LUMO (23%), H-1->L+1 (62%)
302.20882614	0.0028	HOMO->L+5 (37%), HOMO->L+6 (42%)
300.087600475	0.1886	H-5->LUMO (59%), H-1->L+1 (16%)
296.478139153	0.0956	H-7->LUMO (22%), H-6->LUMO (46%)
293.203880746	0.0063	H-8->LUMO (22%), HOMO->L+5 (28%), HOMO->L+6 (31%)
290.633363835	0.0014	H-7->LUMO (32%), H-6->LUMO (12%), HOMO->L+7 (40%)
289.473028909	0.0054	H-9->LUMO (58%), H-8->LUMO (26%)
283.658269492	0.0821	H-12->LUMO (13%), HOMO->L+8 (55%)
281.347447155	0.0079	H-10->LUMO (31%), H-2->L+1 (35%), H-1->L+2 (10%)
280.051032283	0.0171	H-9->LUMO (23%), H-8->LUMO (18%), HOMO->L+5 (16%)
279.68462218	0.0045	H-8->LUMO (14%), H-7->LUMO (18%), HOMO->L+7 (44%)
276.756608434	0.0152	H-10->LUMO (41%), H-2->L+1 (42%)
273.267490274	0.0091	H-10->LUMO (13%), H-1->L+2 (80%)
271.871311754	0.0045	H-11->LUMO (84%)
266.174738111	0.4469	H-12->LUMO (25%), H-3->L+1 (18%), HOMO->L+8 (14%)
261.244849264	0.0137	H-13->LUMO (14%), H-12->LUMO (12%), H-4->L+1 (20%), H-3->L+1 (27%)
255.827403871	0.0117	H-12->LUMO (12%), H-4->L+1 (58%), H-3->L+1 (15%)
252.889617991	0.0029	H-13->LUMO (46%), H-3->L+1 (18%), HOMO->L+9 (18%)
250.422526787	0.0032	H-5->L+1 (85%)
250.068965333	0.0252	H-2->L+2 (60%), H-1->L+3 (22%)

248.355821105	0.0194	H-6->L+1 (13%), H-2->L+2 (16%), H-1->L+3 (56%)
247.009987274	0.0178	H-7->L+1 (18%), H-6->L+1 (39%), H-1->L+3 (10%)
246.401273922	0.004	H-7->L+1 (13%), H-1->L+4 (62%)
246.156672912	0.0044	H-8->L+1 (16%), H-6->L+1 (15%), H-1->L+4 (26%), H-1->L+5 (10%)
245.23645195	0.0024	H-8->L+1 (21%), H-7->L+1 (27%), H-1->L+7 (10%)
242.71126013	0.0232	H-9->L+1 (67%), H-8->L+1 (19%)
240.942502647	0.0534	H-1->L+5 (27%), H-1->L+6 (55%)
239.624655519	0.0026	HOMO->L+9 (57%)
238.087744623	0.0008	H-5->L+2 (22%), H-2->L+4 (27%)
236.963787723	0.0037	H-10->L+1 (13%), HOMO->L+10 (50%)
235.962608504	0.0044	H-4->L+3 (13%), H-2->L+3 (22%)
234.003082086	0.0071	H-3->L+2 (26%), H-1->L+5 (12%), H-1->L+8 (20%)
233.694335983	0.0434	H-10->L+1 (13%), H-4->L+2 (16%), H-3->L+2 (25%), HOMO->L+10 (10%)
233.346871082	0.0115	H-10->L+1 (56%)
231.331056445	0.0111	H-11->L+1 (20%), H-4->L+2 (42%)
230.608200677	0.0432	H-11->L+1 (24%), H-9->L+1 (10%), H-8->L+1 (14%), H-1->L+5 (16%), H-1->L+8 (14%)
230.209987582	0.0411	H-7->L+1 (16%), H-1->L+7 (50%)
229.532347845	0.024	H-12->L+1 (14%), H-11->L+1 (25%), H-4->L+2 (13%), H-1->L+8 (15%)
226.314604651	0.0073	H-12->L+1 (10%), H-11->L+1 (14%), H-7->L+2 (11%), H-6->L+2 (21%), H-1->L+8 (12%)
224.637531956	0.0636	H-5->L+2 (39%), H-2->L+4 (28%)
222.804810704	0.004	H-12->L+1 (36%), H-7->L+2 (11%), H-6->L+2 (14%)

Table S16. Major transitions were calculated using TD-DFT studies of **6b**.

**Wavelength (nm)**	**Osc. Strength**	**Major contribs**
497.3492	1.3565	HOMO->LUMO (97%)
435.0781	0	H-1->LUMO (96%)
385.0321	0.3356	H-2->LUMO (96%)
378.7165	0	HOMO->L+1 (94%)
341.432	0.1095	H-1->L+1 (93%)
329.1499	0	H-3->LUMO (88%)
312.3578	0	H-2->L+1 (88%)
312.1062	0.006	H-4->LUMO (77%)
310.5039	0	HOMO->L+3 (92%)
306.3153	0.0435	HOMO->L+2 (48%), HOMO->L+5 (43%)
305.4625	0	H-5->LUMO (88%)
303.2659	0	HOMO->L+4 (83%)
299.4209	0.0061	H-6->LUMO (93%)
296.9539	0	H-7->LUMO (76%)
296.9112	0.0104	HOMO->L+5 (12%), HOMO->L+6 (79%)
293.6135	0.094	HOMO->L+2 (30%), HOMO->L+5 (37%), HOMO->L+6 (15%)
281.5647	0.0261	H-8->LUMO (84%)
277.4751	0	H-9->LUMO (76%), H-1->L+2 (18%)
275.6613	0	H-9->LUMO (14%), H-1->L+2 (70%)

274.2467	0.0009	H-1->L+3 (89%)
273.2916	0.121	H-3->L+1 (70%)
270.832	0.0043	H-1->L+4 (86%)
269.971	0	H-10->LUMO (22%), H-1->L+5 (65%)
267.3341	0	H-10->LUMO (55%), H-1->L+5 (23%)
264.9461	0	H-2->L+3 (14%), H-1->L+6 (74%)
262.4837	0.0116	H-12->LUMO (14%), H-11->LUMO (67%)
260.8711	0.2725	H-12->LUMO (31%), H-11->LUMO (12%), H-3->L+1 (11%), H-2->L+2 (22%)
259.0939	0	H-10->LUMO (10%), H-7->LUMO (11%), H-4->L+1 (53%)
255.9383	0	H-13->LUMO (12%), H-2->L+3 (45%), HOMO->L+7 (21%)
255.5216	0.223	H-12->LUMO (10%), H-2->L+2 (30%), HOMO->L+8 (21%)
255.0643	0	H-13->LUMO (12%), H-2->L+3 (32%), H-1->L+6 (14%), HOMO->L+7 (26%)
254.7341	0.0767	H-2->L+2 (25%), H-2->L+5 (25%), HOMO->L+8 (26%)
252.5085	0	H-2->L+4 (78%)
250.701	0.0449	H-5->L+1 (50%), H-2->L+5 (34%)
250.2305	0.0383	H-5->L+1 (30%), H-2->L+5 (31%), HOMO->L+8 (18%)
247.8593	0	H-6->L+1 (89%)
247.0248	0.0083	H-2->L+6 (88%)
245.5667	0	H-13->LUMO (32%), H-4->L+1 (26%), HOMO->L+7 (23%)
243.1063	0.0199	H-12->LUMO (15%), H-7->L+1 (58%), HOMO->L+8 (15%)
236.5614	0	H-8->L+1 (88%)
234.4899	0.0728	H-1->L+7 (53%), HOMO->L+8 (10%)
233.9633	0	H-2->L+7 (10%), H-1->L+8 (50%)
233.6899	0.0601	H-9->L+1 (85%)
232.2671	0	HOMO->L+9 (82%)
231.6509	0.033	H-3->L+4 (24%), HOMO->L+10 (34%)
231.2491	0	H-3->L+2 (37%), H-3->L+5 (20%)
230.6382	0.0152	H-3->L+3 (70%), HOMO->L+10 (11%)
230.21	0.0104	H-3->L+3 (20%), H-3->L+4 (20%), HOMO->L+10 (16%)
228.673	0	H-5->L+5 (10%), H-3->L+2 (35%)
226.0753	0.0169	H-10->L+1 (79%)

Table S17. Major transitions were calculated using TD-DFT studies of **6c**.

**Wavelength (nm)**	**Osc. Strength**	**Major contribs**
538.312751877	1.3392	HOMO->LUMO (99%)
417.160233546	0.0018	H-1->LUMO (63%), HOMO->L+1 (36%)
400.284732396	0.0624	H-1->LUMO (34%), HOMO->L+1 (61%)
369.06647917	0.0705	H-2->LUMO (91%)
348.573738402	0.2508	HOMO->L+2 (87%)
335.038083047	0.0177	H-3->LUMO (91%)
330.633331588	0.0468	H-4->LUMO (27%), H-1->L+1 (68%)
325.956813135	0.476	H-4->LUMO (59%), H-1->L+1 (25%)
318.676278754	0.0011	HOMO->L+3 (94%)

315.915489508	0.0034	HOMO->L+4 (94%)
311.299068525	0.026	H-5->LUMO (56%), HOMO->L+5 (25%)
307.058777087	0.0331	H-5->LUMO (19%), HOMO->L+5 (53%), HOMO->L+6 (10%)
304.786727826	0.005	H-6->LUMO (63%), HOMO->L+5 (13%)
302.481624368	0.0079	H-7->LUMO (22%), H-2->L+1 (60%)
301.650024359	0.0204	H-7->LUMO (61%), H-2->L+1 (26%)
292.091768587	0.0101	H-8->LUMO (80%)
290.851536577	0.0102	H-9->LUMO (71%), H-1->L+2 (12%)
287.779850549	0.1016	H-12->LUMO (14%), HOMO->L+6 (51%)
285.987574129	0.0041	H-9->LUMO (10%), H-1->L+2 (77%)
279.564799685	0.0182	H-12->LUMO (20%), H-10->LUMO (45%)
277.9852313	0.0734	H-11->LUMO (13%), H-3->L+1 (65%)
274.331658396	0.0077	H-11->LUMO (57%), H-10->LUMO (11%)
271.228983663	0.2192	H-11->LUMO (19%), H-10->LUMO (19%), H-4->L+1 (16%), H-3->L+1 (10%)
269.753694383	0.0389	H-12->LUMO (14%), H-4->L+1 (61%), HOMO->L+7 (10%)
264.155856938	0.0526	H-2->L+2 (59%)
262.86215577	0.0067	H-13->LUMO (25%), H-2->L+2 (10%), H-1->L+3 (15%), HOMO->L+7 (15%), HOMO->L+8 (10%)
262.161827358	0.0066	H-5->L+1 (12%), H-1->L+3 (49%), HOMO->L+8 (10%)
261.514855541	0.0007	H-1->L+3 (27%), HOMO->L+7 (39%)
259.995791331	0.0065	H-1->L+4 (83%)
259.007276133	0.0873	HOMO->L+8 (56%)
256.749208971	0.0199	H-6->L+1 (27%), H-5->L+1 (45%)
253.235688342	0.0526	H-1->L+5 (79%)
252.49306169	0.0045	H-7->L+1 (57%), H-6->L+1 (20%)
250.84812247	0.0059	H-13->LUMO (11%), H-7->L+1 (29%), H-6->L+1 (24%), H-5->L+1 (12%)
246.151785845	0.0023	H-3->L+2 (22%), H-1->L+6 (51%)
245.382059121	0.0394	H-9->L+1 (33%), H-8->L+1 (48%)
244.347161097	0.0037	H-3->L+2 (61%), H-1->L+6 (18%)
243.77065534	0.0069	H-9->L+1 (49%), H-8->L+1 (30%)
242.450219039	0.0069	H-2->L+3 (68%)
241.722282251	0.0049	H-2->L+4 (60%)
237.699756542	0.1022	H-4->L+2 (81%)
236.611055367	0.0046	H-10->L+1 (15%), HOMO->L+9 (40%), HOMO->L+10 (23%)
235.317706142	0.0076	H-10->L+1 (21%), H-2->L+5 (10%), HOMO->L+9 (30%), HOMO->L+10 (18%)
233.202033277	0.0088	H-11->L+1 (15%), H-2->L+5 (51%)
232.94352844	0.0003	H-11->L+1 (55%)
232.175788866	0.0416	H-2->L+6 (23%), H-1->L+7 (10%)
231.798147271	0.0094	H-11->L+1 (12%), H-7->L+2 (22%), H-2->L+4 (13%)
231.434691653	0.0017	H-10->L+1 (15%), H-2->L+6 (14%), HOMO->L+10 (18%)
230.496733616	0.0021	H-8->L+2 (14%), H-4->L+3 (14%)
228.348668433	0.0086	H-12->L+1 (22%), H-5->L+2 (25%), H-2->L+6 (12%)

References:

[1] M. Fischer, J. Georges, *Chem. Phys. Lett.* 1996, **260**, 115–118.

[2] S. Uchiyama, Y. Matsumura, A. P. de Silva, K. Iwai, *Anal. Chem.* 2004, **76**, 1793–1798.

[3] J. R. Lakowicz, B. R. Masters, *J. Biomed. Opt.* 2008, **13**, 029901.

[4] L. J. Bourhis, O. V. Dolomanov, R. J. Gildea, J. A. K. Howard, H. Puschmann, *Acta Crystallogr. A Found. Adv.* 2015, **71**, 59–75.

[5] O. V. Dolomanov, L. J. Bourhis, R. J. Gildea, J. A. K. Howard, H. Puschmann, *J. Appl. Crystallogr.* 2009, **42**, 339–341.

[6] S. Gilb, K. Jacobsen, D. Schooss, F. Furche, R. Ahlrichs, M. M. Kappes, *J. Chem. Phys.* 2004, **121**, 4619–4627.

[7] M.J. Turner, J.J. McKinnon, S.K. Wolff, D.J. Grimwood, P.R. Spackman, D. Jayatilaka, M.A. Spackman, CrystalExplorer17, University of Western Australia, 2017. http://hirshfeldsurface.net.

[8] M. Yu, Y. Zhang, Y.-Q. Pan, L. Wang, *Inorganica Chim. Acta,* 2020, **509**, 119701.

--00--
